# Virulent Parasites Emerge in Hosts With Rising Temperatures

**DOI:** 10.1111/gcb.71082

**Published:** 2026-09-08

**Authors:** Tobias E. Hector, Julia M. Kreiner, James C. Forward, Kim L. Hoang, Emily J. Stevens, Serena Johnson, Jingdi Li, Kayla C. King

**Affiliations:** ^1^ Department of Biology University of Oxford Oxford UK; ^2^ Biodiversity Research Centre University of British Columbia Vancouver British Columbia Canada; ^3^ Department of Ecology & Evolution University of Chicago Chicago Illinois USA; ^4^ Department of Microbiology & Immunology University of British Columbia Vancouver British Columbia Canada; ^5^ Emory School of Medicine Emory University Atlanta Georgia USA; ^6^ School of Life Sciences Keele University Staffordshire UK; ^7^ Department of Zoology University of British Columbia Vancouver British Columbia Canada

**Keywords:** climate warming, experimental evolution, host–parasite, population genomics, thermal mismatch, virulence

## Abstract

Climate change is increasing the risk of emerging parasites. However, whether more virulent variants will spread during climate‐driven outbreaks remains unclear. Here, we aimed to explore the short‐term trajectory of parasite evolution—at the phenotypic and genomic scales—across environmentally relevant temperatures in a thermally mismatched host–parasite interaction. We experimentally evolved a wild parasitic bacterium (*Leucobacter musarum*), across the thermal range (20°C–30°C) and extremes (35°C) of Cabo Verde—the site of field collection—in a *Caenorhabditis elegans* host strain. Starting from a single bacterial isolate, we then tracked phenotypic and *de novo* genomic changes that arose across replicate populations following ten passages of experimental evolution. We found that at 25°C, warm for the host but an average temperature for the parasite, host‐mediated selection favoured higher virulence and genomic diversification by the end of the experiment. At hot temperatures, towards the limit of host survival, virulence was maintained across all parasite populations. Parasites evolved at hot temperatures also displayed a latent virulence boost, deadlier once hosts experienced a heatwave. Patterns of molecular evolution were constrained to parallel changes in fewer loci at extreme temperatures. Our findings suggest that shifting environmental temperatures will leave phenotypic and genomic signatures on evolving parasites.

## Graphical abstract

Using experimental evolution we explored the short‐term trajectory of parasite evolution—at the phenotypic and genomic scales—across environmentally relevant temperatures in a thermally mismatched host–parasite interaction. Our findings suggest that shifting environmental temperatures will leave phenotypic and genomic signatures on evolving parasites.

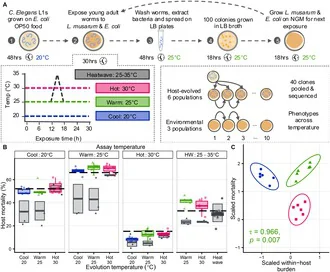

## 1 Introduction

Climate change is associated with outbreaks of both established and emerging infectious diseases (Mora et al. 2022; Mahon et al. 2024). As temperate regions warm and become suitable for parasites from lower latitudes (Gehman et al. 2018; Ryan et al. 2019; Cohen et al. 2020), there are heightened risks of parasites from warmer regions becoming established within host populations in historically cooler regions (Carlson et al. 2017; Pfenning‐Butterworth et al. 2024). Mismatches between host and parasite thermal tolerances are predicted to exacerbate infections when warm temperatures are favourable for the parasite (Cohen et al. 2020). Yet, as parasites colonise and establish within a species, they cannot fully exploit host resources. This suboptimal environment forces parasites to adapt quickly, sometimes losing or gaining virulence (i.e., harm caused to infected hosts) to improve ongoing transmission (Alizon et al. 2009; Gandon et al. 2013; Geoghegan and Holmes 2018). The dynamics and outcomes of parasite evolution are understudied in most wild, emerging disease systems (Bolker et al. 2010; Tardy et al. 2019). Local and global outbreaks of zoonotic diseases have revealed an urgent need to forecast evolutionary trajectories of virulence (Day, Gandon, et al. 2020; Alizon and Sofonea 2021), particularly in an era now defined by warming and extreme temperatures (Pörtner et al. 2022).

It remains unclear to what extent temperature can shape parasite evolution (Laine 2008; Schampera et al. 2022; Hector et al. 2023; Greenrod et al. 2025). When not at evolutionary equilibrium (Anderson and May 1982; Frank 1996), virulence evolution can become decoupled from replication and transmission (Bull and Ebert 2008; Visher et al. 2021), particularly if initial parasite adaptation occurs in traits not directly involved in host exploitation (e.g., immune evasion) (Sasaki et al. 2022). Warming might favour high virulence if parasite fitness is not optimal in the new thermal environment (Alizon and Sofonea 2021) or if heat stress enhances host tolerance (André et al. 2003). An alternative trajectory might arise when a host's ability to withstand heat is impaired by infection (Hector et al. 2021, 2022). Heat stressed hosts being sicker might shorten the infectious period—an outcome predicted to either favour less virulent variants (Day, Parsons, et al. 2020; Hector et al. 2023) or constrain adaptive evolution due to population bottlenecks (Wahl and Gerrish 2001). Temperature is also a key selective force on parasite free‐living stages. Warming can extend the seasonal window for parasite growth, directly contributing to their development and between‐host transmission (Hernandez et al. 2013; Molnár et al. 2013; Vezzulli et al. 2013), but can reduce the survival of some free‐living stages in the environment (Altizer et al. 2013; Molnár et al. 2013). Virulence evolution will ultimately depend on the relative strength and direction of selection, as well as the trade‐offs involved in adapting to the within‐ and between‐host environments as temperatures climb (Dutta et al. 2021; Pandey et al. 2022).

**FIGURE 1:**
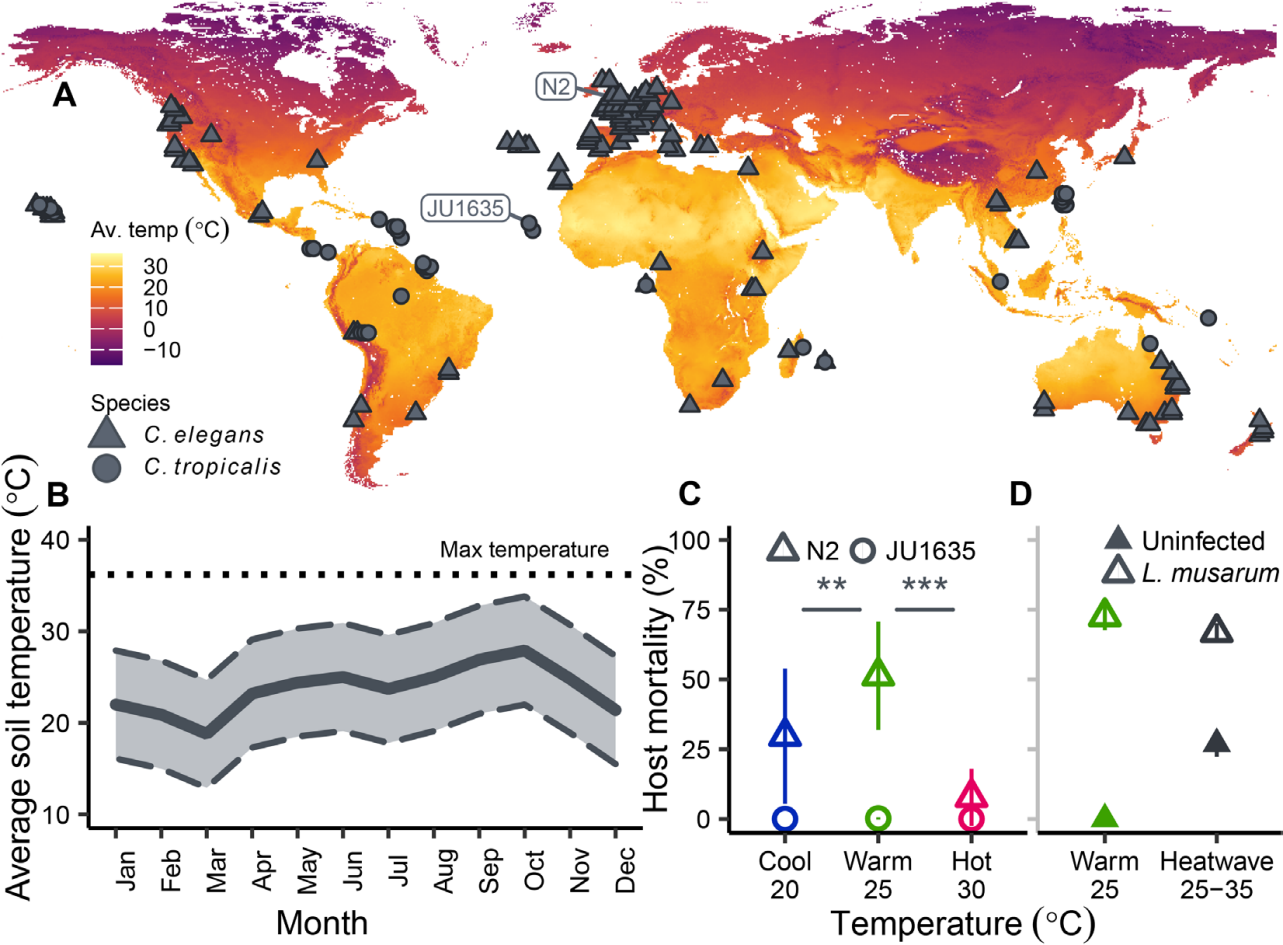
Temperature natural history of *Caenorhabditis* nematodes and the associated virulence of bacterial parasite *Leucobacter musarum*. (A) Known species distributions of the natural host ( *C. tropicalis* ) and recently experimentally established host ( *C. elegans* ) [data from (Kiontke et al. 2011; Crombie et al. 2023)]. Map colours represent annual average soil surface temperatures (Lembrechts et al. 2022). Collection locations for *C. tropicalis* JU1635 from which *L. musarum* was isolated and *C. elegans* N2 hosts are labelled. (B) Predicted average soil temperatures (at 0.5 cm depth) for each month (solid line), average diurnal variation (dashed lines), and the average maximum soil temperature (dotted line) from the location *L. musarum* was isolated [data from (Lembrechts et al. 2022)]. (C) *C. elegans* N2 (triangles) and *C. tropicalis* JU1635 (circles) mortality when exposed to the ancestral *L. musarum* clone at three ecologically relevant constant temperatures (see Methods). Uninfected nematodes have negligible mortality across this thermal range. Stars show significant pairwise comparisons between both 20°C (Dunnet's post hoc test, *z* = −3.485, *p* = 0.001 **) and 30°C (Dunnet's post hoc test, *z* = −11.56, *p* = < 0.001 ***) relative to 25°C for infected *C. elegans* N2 hosts (betabinomial ANOVA, *χ* ^2^ = 135.57, df = 2, *p* = < 0.001). Mean values (± 1 SD) are shown ( *C. elegans* *n* = 10 per temperature, *C. tropicalis* *n* = 5 per temperature). (D) Simulated heatwaves (4‐h ramp between 25°C and 35°C) cause mortality in uninfected *C. elegans* populations (Tukey's post hoc test comparing uninfected mortality at 25°C and heatwave, *t* = 10.252, *p* = < 0.001, *n* = 4). During infection, mortality is high across both constant 25°C and heatwave temperatures (Tukey's post hoc test comparing infected mortality at 25°C and heatwave, *t* = −2.165, *p* = 0.051, *n* = 4). Data in (C, D) were collected as part of this study in independent assays and absolute values are not directly comparable.

To test these predictions, we experimentally evolved a wild, facultative bacterial parasite (*Leucobacter musarum*) in a *Caenorhabditis elegans* nematode host strain at ecologically relevant temperatures. *Leucobacter* species have a global distribution and are frequently found as animal associates (including with *Caenorhabditis* nematodes) in natural and agricultural systems (Bates et al. 2021). The parasite *L. musarum* was isolated from the congener host *C. tropicalis* in rotten banana pseudostems in Cabo Verde (Figure 1A) (Hodgkin et al. 2013; Clark and Hodgkin 2015). Cabo Verde is a tropical archipelago in the central Atlantic Ocean, where average surface soil temperatures are around 25°C, ranging between 19°C and 28°C, with maximum average soil temperatures as high as 36°C (Figure 1B) (Lembrechts et al. 2022). The sympatric host and parasite are both well adapted to these warm environmental temperatures. Both in vitro and within‐host parasite replication peak between 25°C and 30°C (Figure S1) (See also Will et al. 2025), and *C. tropicalis* can maintain reproduction across much of this thermal range (Poullet et al. 2015).

In contrast, *C. elegans* are generally adapted to temperate climates. Fitness is maximised between 15°C and 20°C, thermal stress responses are active at 25°C, and prolonged exposure above 27°C causes sterility (Begasse et al. 2015; Gouvêa et al. 2015). This species is not present in Cabo Verde despite rare occurrences at high altitudes in other tropical regions (Figure 1A) (Frézal and Félix 2015; Braendle and Paaby 2024), and this host–parasite pairing represents a relatively recent evolutionary interaction exhibiting clear thermal mismatch. Transmission of *L. musarum* can occur via direct host–host contact and acquisition from the environment, with infection and virulence comparable across domesticated and wild‐type *C. elegans* strains (Bates et al. 2021). During infection in *C. elegans* , *L. musarum* attaches to the cuticle before infiltrating the host via the rectum, rapidly inducing systemic infection, reduced fecundity and death (Figure 1C) (See also Hodgkin et al. 2013). As with wild emerging parasites, infection outcomes are less severe in the sympatric host with a longer history of infection and opportunity for an evolutionary response (Figure 1C) (Bolker et al. 2010; Geoghegan and Holmes 2018; Li et al. 2024; Sauer et al. 2024).

**FIGURE 2:**
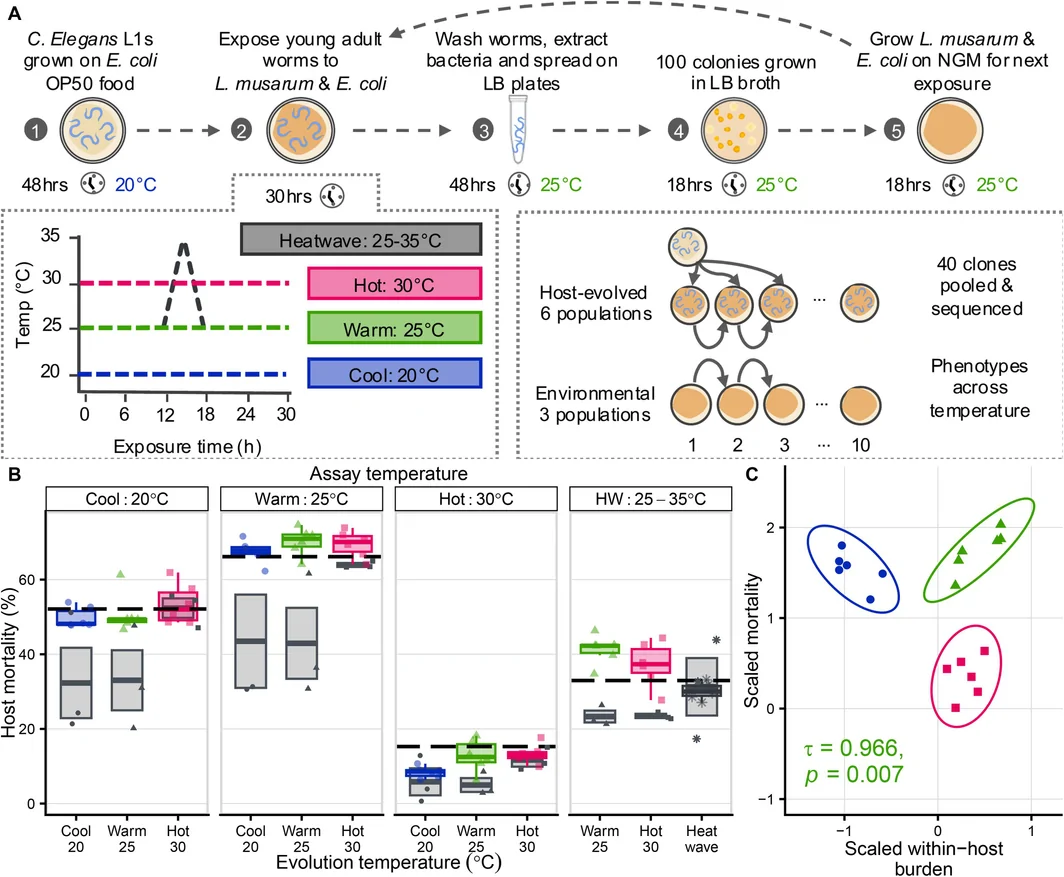
Experimental evolution reveals the impact of temperature on parasite evolution. (A) visual overview of experimental evolution protocol, which was repeated for a total of ten passages. Parasite populations only experienced differences in temperature during experimental infections, all other in vitro culturing took place at either 25°C (for *L. musarum*), 20°C (for *C. elegans* ) or 30°C (for *E. coli* OP50 food source). We included three ecologically relevant constant temperatures (cool: 20°C, warm: 25°C and hot: 30°C), and a ‘heatwave’ treatment (HW). During heatwaves, temperatures were quickly ramped from 25°C to 35°C (~0.1°C/min) and then back to 25°C over a 4‐h period. These peak temperatures are beyond the thermal maxima for both the host and parasite (Figure S3). (B) Mortality (%) of *C. elegans* hosts exposed to parasite populations (Passage 10) at each temperature (facets show the assay temperature at which mortality was measured, and the x‐axis shows the experimental evolution temperature treatment) in a factorial common garden experiment. Host‐evolved parasite populations are shown with coloured boxplots and points, and grey boxes and points show the mean and standard deviation of the environmental control populations. Dashed horizontal lines show the mean ancestral phenotype. Data for other treatment combinations in Figure S4. (C) Relationship between within‐host bacterial burden and host mortality during assay infections at 25°C for host‐evolved populations (population means centred relative to their respective environmental controls and scaled to allow comparison, colours correspond to those in B), showing Kendall's rank correlation (*n* = 6, *τ* = 0.966, *z* = 2.678, *p*‐value = 0.007) across 25°C populations (green).

To investigate short‐term parasite evolution, we experimentally passaged parasite populations independently across 10 generations of nematode populations and four temperature regimes (Figure 2A). Temperatures covered the thermal range (20°C–30°C) and extremes (35°C) of Cabo Verde. They also spanned the thermal mismatch between host and parasite species. Environmental (host‐free) controls were conducted alongside at each temperature by passaging the parasite in the absence of any hosts. The host population remained evolutionary static throughout the experiment. We carried out phenotypic assays of host mortality upon infection (metric for parasite virulence) and load (metric for parasite replication) across treatments. We then used shotgun sequencing of pools of colonies to measure evolutionary changes in the genomic composition of *L. musarum* populations and to identify molecular targets of selection.

## 2 Materials and Methods

### 2.1 Host–Parasite System

We used a natural bacterial parasite of *Caenorhabditis* nematodes, *Leucobacter musarum* sp. nov. subsp. *musarum* subsp. nov. strain CBX152T (Hodgkin et al. 2013). *Leucobacter musarum* is a gram‐positive bacterium isolated from infected *C. tropicalis* nematodes (JU1635 strain), collected from rotting banana trunks in the Republic of Cabo Verde (Kiontke et al. 2011) (Figure 1). While the *Leucobacter* genus is globally ubiquitous, often associated with animal microbiomes, an analysis of *Leucobacter* microbiome OTUs in 13,715 samples from 163 published studies spanning all continents except Antarctica did not find the Cabo Verde subspecies of *L. musarum* associated with *C. elegans* (Bates et al. 2021). *C. elegans* has also never been found in Cabo Verde (Frézal and Félix 2015). For our experimental context (short‐term rapid evolution) we therefore assume that *C. elegans* does not have a recent evolutionary history prior to isolation in the lab (see below) with the sub‐species (*Leucobacter musarum* sp. *nov. subsp. musarum subsp. nov*. strain CBX152T) used here.

In this study, we used simultaneous hermaphroditic *C. elegans* N2 congener host strain (*Caenorhabditis* Genetics Centre, University of Minnesota, Minneapolis, MN), originally isolated from Bristol, UK. Although N2 has undergone domestication‐associated phenotypic and genomic changes affecting traits such as behaviour, stress responses and parasite resistance (Sterken et al. 2015), it also shares many characteristics with wild isolates. N2 has been domesticated under laboratory conditions at 15°C–20°C, a thermal history that aligns with optimal temperatures for most wild‐type *C. elegans* strains (Petrella 2014; Poullet et al. 2015; Frézal et al. 2018). Global *C. elegans* genomic diversity is characterised by generally low levels of variation, and the N2 strain displays intermediate levels of genomic divergence (Figure S2). Despite its domestication history, the N2 strain shares common haplotypes and environment‐associated alleles with the majority of wild‐type isolates from Europe (Andersen et al. 2012; Evans et al. 2017; Lee et al. 2021), and exhibits environmental responses within the range of natural variation (Ashe et al. 2013; Balla et al. 2015; Polk et al. 2025). Gene expression is also known to vary across domesticated and wild‐type isolates, yet a strong positive correlation in gene expression levels in response to gram‐positive bacteria has been found between N2 and the highly diverged Hawaiian CB4856 (Lansdon et al. 2022). The route of *L. musarum* infection is also identical across hosts' species and host mortality is broadly comparable between N2 and geographically distinct wild *C. elegans* isolates (Bates et al. 2021), suggesting that key aspects of host susceptibility are ecologically relevant despite domestication. N2 represents a simplified experimental model of a recently invaded host species with a distinct thermal history from the parasite, where the route and progression of infection remain comparable with the sympatric host, but the outcomes of infection are more severe for the new host species (Figure 1C). Combining a domesticated host with a wild parasite may however influence eco‐evolutionary responses (Malinski et al. 2025), and our simplified system cannot capture the full ecological and genetic diversity of natural host–parasite interactions.

### 2.2 Preparation of Host Population Stocks and Ancestral Bacteria

Nematode maintenance followed standard protocols (Stiernagle 2006) (See Appendix S1 for detailed protocols for each procedure mentioned below). Prior to our evolution experiment, a population of *C. elegans* (N2 strain) was defrosted from glycerol stocks and grown on nematode growth medium (NGM) in 9 cm petri plates seeded with *Escherichia coli* OP50 (hereafter referred to as OP50; *Caenorhabditis* Genetics Centre, University of Minnesota, Minneapolis, MN). This food bacterium (OP50) was grown at 30°C shaking at 200 rpm overnight in LB broth, with 100 μL of culture subsequently spread onto each NGM plate and incubated overnight at 30°C. Plates of *C. elegans* containing gravid females were ‘bleached’ using a sodium hypochlorite solution to surface sterilise eggs and synchronise the population. Aliquots of hatched L1 worms were subsequently frozen in sterile worm freezing medium at −80°C. These frozen worm stocks were used throughout the evolution experiment as an evolutionary static host population.

*Leucobacter musarum* was isolated by M‐A. Fèlix & J. Hodgkin (Hodgkin et al. 2013; Clark and Hodgkin 2015). Briefly, infected *C. tropicalis* nematodes were added to *E. coli* lawns for 12 h at room temperature, allowing the transfer of surface bacteria to the plate. The *C. tropicalis* were then removed before adult *C. elegans* N2 worms were added and propagated for ~8 generations at 25°C and then transferred to bare NGM plates. After removing the worms from the NGM plates, they were incubated at 30°C for 2–3 days. A colony was picked and grown in liquid LB culture at 30°C to stationary phase and frozen in 50% glycerol at −80°C. From this point on, all in vitro culturing of *L. musarum* took place at 25°C to best match the average environmental conditions, with the aim of minimising strong thermal selection during these steps. Bacteria were streaked from frozen stocks onto LB agar in a 9 cm petri dish and incubated at 25°C for 48 h until distinct colonies were visible. A single colony was picked into 8 mL LB broth in a 15 mL falcon tube and grown overnight at 25°C shaking at 150 rpm. This overnight culture represented our ‘ancestral’ *L. musarum*. A portion was frozen at −80°C in 50% glycerol, and the remainder used to commence the first passage of the evolution experiment (Figure 2A).

### 2.3 Experimental Evolution

We passaged *L. musarum* under four temperatures (20°C, 25°C, 30°C and 25°C–35°C heatwave) and two infection treatments (hosts present and environmental [no‐host] controls) (Figure 2A). At each temperature, we independently passaged six replicate host‐evolved populations (24 populations total), and three replicate environmental control populations (*n* = 12). Our unit of biological replication was each independently evolving bacterial population. We ran the experiment for ten passages, equating to ten host generations. All in vitro culturing of *L. musarum* was conducted at a common temperature (25°C), OP50 at 30°C, and all nematode maintenance at 20°C (optimal for *C. elegans* ), to minimise and standardise laboratory selection.

For the experimental exposure plates, we standardised the ancestral *L. musarum* culture to an optical density (OD_630_) of 0.2, and an overnight culture of OP50 to OD_630_ of 0.4. The two bacterial cultures (parasite and food) were then combined with one part *L. musarum* and four parts OP50 (1:4), and 200 μL was spread on 90 mm NGM plates (host‐present treatments), or 200 μL of *L. musarum* alone for environmental (no‐host) control treatments. All plates were incubated overnight at 25°C. It is possible that the parasite and OP50 interacted for a short period of time on the host‐evolved exposure plates during the overnight plate incubation. All other culturing was conducted separately for the two bacteria species. Interactions with OP50 are unlikely to play a major role in parasite evolution. Co‐culture on the exposure plates represents a short period of time (< 20%) during a single passage, particularly given *L. musarum* grows much slower than OP50 (~48 h for colonies to form on solid media, compared with 8–12 h for OP50). The parasites ultimately passaged in the host‐evolved treatments interacted with nematodes longer in the experimental regime. During association with hosts, OP50 is digested while the parasite adheres to the cuticle of the worm and enters the body cavity via the rectum. The distinct spatial separation on the host formed the basis for choosing to grow the environmental control treatments alone during the ‘infection period’. Moreover, OP50 is biofilm‐formation deficient (Arata et al. 2020). Where biofilm formation is induced by bacterial competition, this is usually mediated by the dominant strain (Oliveira et al. 2015) which is OP50, based on the asymmetric doses used here—4 parts OP50 to 1 part *L. musarum*.

Prior to experimental infections, recently defrosted nematodes from our standardised population were surface‐sterilised and age‐synchronised. Eggs were allowed to hatch overnight in M9 media at 20°C. After hatching, L1 larvae were spotted onto lawns of OP50 grown on NGM plates (approx. 2500 L1s per plate) and allowed to develop to young adults at 20°C (approx. 60 h). For experimental parasite exposures, 1000 young adult worms were spotted onto each ‘host‐evolved’ experimental exposure plate and allowed to dry under sterile conditions. All plates were sealed with Parafilm and placed at their respective treatment temperatures for 30 h. All environmental control populations were treated identically except for the addition of nematodes.

After the 30‐h infection period, all plates were taken out of their respective treatment temperatures and kept at room temperature. For each host‐evolved population, 60 worms were picked randomly with a sterile platinum wire into 1.5 mL Eppendorf tubes containing five to ten 0.5 mm zircon beads and filled with 1200 μL M9‐Tx. Once all populations had been picked, worms were centrifuged at 1000 rpm for 1 min, 1000 μL of the supernatant was removed, and 1000 μL sterile M9‐Tx was added. This process was repeated two more times to gently wash worms of excess surface bacteria. After the final wash step (using M9 without Triton‐X), worms were disintegrated in a tissue lyser in 200 μL of M9. Lysed worm solutions were then diluted to 10^−4^ in sterile M9, and 100 μL of this dilution was spread onto a 9 cm LB agar plate using glass beads. For environmental (no‐host) control populations, bacteria were transferred directly onto 9 cm LB petri dishes from the experimental exposure plates using inoculation loops. All plates were subsequently incubated at 25°C for 48 h.

After 48 h an inoculation pick was used to pick 100 colonies (host‐evolved treatments) or a streak taken (environmental controls) from each population into 3 mL LB broth in 15 mL falcons. Tubes were incubated overnight at 25°C shaking at 150 rpm. A portion of each overnight culture was frozen in 50% glycerol at −80°C, and the remainder was used to make exposure plates for the next passage.

### 2.4 Host Survival and Parasite CFU Assays

To assess changes in parasite infection traits, we performed phenotypic assays using populations from passage 10 alongside the ancestral culture. Given the consistency in the selection pressure applied, we predicted selection to be directional. We therefore focussed on the endpoint of the experiment (e.g., Hoang et al. 2024; Stevens et al. 2025), rather than intermediate timepoints as would be necessary for detecting fluctuating selection (e.g., Papkou et al. 2019). In *C. elegans* , mortality was the most relevant/severe virulence trait during infection, and in the evolution experiment, parasite traits causing host mortality were under selection. We therefore focussed on host mortality as our proxy for parasite virulence.

Age‐synchronised young adult nematodes were prepared as described above. Bacterial parasite populations were inoculated directly from frozen stocks into LB broth and grown for 18 h at 25°C. Exposure plates were prepared as described above. For assays we used either 60 mm or 35 mm NGM plates. Approximately 100–200 young adult nematodes were transferred to each exposure plate and then incubated at the appropriate assay temperature. After ~36 h, the number of live and dead nematodes was counted using a dissecting microscope. Worms were scored as dead if they did not respond to being prodded by a platinum wire. For constant temperature assays, we measured mortality on five technical replicates per population and assay temperature combination, and three for heatwave assays.

Colony forming units (CFU), a proxy for infection burden, were measured following a similar protocol to that used during experimental evolution (see above and Appendix S1). After 36‐h experimental infections, 10 worms per technical replicate were randomly picked using a sterile platinum wire into 1.5 mL Eppendorf tubes containing zircon beads and M9‐Tx. Worms were washed three times before being crushed with a tissue lyser. Samples were serially diluted to 10^−4^ and spotted onto LB agar plates and incubated at 25°C for 48‐h. Colonies were then counted and used to calculate the average number of *L. musarum* bacteria per worm. We measured CFUs on three technical replicates per population and assay temperature combination.

### 2.5 In Vitro Growth Assays

The ancestral isolate and evolved populations were initially cultured from frozen populations into LB broth at 25°C overnight. Subsequently, the OD_600_ of the bacterial samples was standardised and transferred into wells of a transparent 96‐well plate. The optical density was recorded using a BioTek Synergy H1 microplate spectrophotometer (Agilent, Santa Clara, CA). Between the OD measurements, which occurred approximately every 10 min for 24 h, orbital shaking was performed to ensure uniform cell distribution. Growth metrics were mathematically computed using the ‘gcplyr’ R package (Blazanin 2024).

### 2.6 Biofilm Assays

Biofilm formation was assessed using a crystal violet staining method adapted for 96‐well microtiter plates. Bacterial cultures were grown in 96‐well plates with a total volume of 200 μL per well. The plates were incubated under the desired experimental temperature for 24 h. After incubation, the culture supernatants were carefully removed and then gently washed twice with 200 μL of phosphate‐buffered saline (PBS, pH 7.4) to remove non‐adherent cells. To stain the biofilms, 100 μL of 0.1% (w/v) crystal violet solution was added to each well. The plates were incubated at room temperature for 30 min to allow the dye to bind to the biofilm matrix. Following staining, the crystal violet solution was carefully removed by pipetting, and the wells were washed twice with PBS to eliminate excess unbound stain. The bound crystal violet was resolubilised by adding 100 μL of 33% (v/v) acetic acid to each well. The plates were then placed on a shaker and agitated gently at room temperature for 20 min to ensure complete dissolution of the dye from the biofilm matrix into the solution. The optical density (OD_575_) of the solubilised crystal violet in each well was measured using a microplate reader. The absorbance values correspond to the amount of biofilm formed by the bacterial cultures under the tested conditions. Six technical replicates were measured per population/treatment combination.

### 2.7 DNA Extraction and Pool Sequencing

Frozen population samples were streaked onto LB agar plates and incubated for 48 h at 25°C when individual colonies could be identified. Forty colonies were picked and grown separately in LB broth at 25°C for 48 h, then the OD_600_ of each single colony culture was standardised before pooling them into one tube to perform DNA extraction. We extracted genomic DNA using a DNeasy Blood and Tissue Kit (Qiagen). Briefly, we pelleted 1 mL of each bacterial sample, removed the supernatant, then resuspended in 180 μL of enzymatic lysis buffer (20 mM Tris–HCl, 2 mM EDTA, 1.2% Triton, and 3.6 mg lysozyme). Samples were incubated at 56°C for 30 min with occasional vortexing. We added 25 μL Proteinase K and 200 μL Buffer AL to each sample, then incubated them at 56°C for 30 min to 1 h. After vortexing for 15 s, we added 200 μL of 100% ethanol and vortexed briefly before transferring to spin columns. We then followed the manufacturer's instructions for washing and eluting DNA. We quantified DNA concentration using a Qubit fluorometer. We assessed DNA purity and integrity using a Nanodrop and gel electrophoresis, respectively.

### 2.8 Ancestral Assembly and Variant Calling

Ancestral assembly was carried out by Xuan Liu at the University of Liverpool Centre for Genomic Research. Initial processing and quality assessment of the sequence data was performed using an in‐house pipeline developed by Dr. Richard Gregory. Briefly, base calling and de‐multiplexing of indexed reads was performed by CASAVA version 1.8.2 (Illumina). The raw FASTQ files were trimmed to remove Illumina adapter sequences (option ‘‐O 3’) using Cutadapt version 1.2.1 (Martin 2011). The reads were further trimmed to remove low quality bases, using Sickle version 1.2 with a minimum window quality score of 20. After trimming, reads shorter than 20 bp were removed. Trimmed reads files were deposited in the NCBI Sequence Read Archive (SRA) under BioProject: PRJNA1208452 (http://www.ncbi.nlm.nih.gov/bioproject/1208452). The ancestral isolate was assembled using SPAdes (Bankevich et al. 2012) and was assessed using BUSCO (Seppey et al. 2019). The assembly was further annotated using Prokka (Seemann 2014). R1/R2 reads from each evolved sample were mapped to the ancestral assembly using BWA mem version 0.7.5a (Li 2013) with default parameters, removing reads with a mapping quality lower than 10. Duplicate reads arising from PCR amplification were identified and filtered to retain only a single representative, using the Picard ‘MarkDuplicates’ tool, version 1.85 (http://picard.sourceforge.net/). In order to quantify putative functional effects of *de novo* mutations throughout our evolution experiment, we initially called SNPs using the Freebayes (Garrison and Marth 2012) using default settings (except ‘ploidy = 40’ was set for population samples, and ‘QUAL > 20’). We then annotated variants using SnpEFF (version 4.3) (Cingolani et al. 2012).

### 2.9 Population Genomic Analysis

To quantify genomic responses over the course of the evolution experiment, we called the frequency of *de novo* mutations in sequenced reads at the experimental endpoint for the 36 distinct pools (parasite populations). Note that we are able to ascribe all SNPs as *de novo* mutations, as the reference genome to which all populations were aligned was generated from the ancestor used to start the experiment. As sequence reads represent pools of clonal populations in each experimental and control treatment, we leveraged a highly efficient recoding of the popular pipeline for experimental evolution PoPoolation2 (v 1.201) (Kofler et al. 2011) called Grenedalf (v 0.6.2) (Czech et al. 2024). We used the ‐‐frequency command to produce a table of *de novo* mutation frequencies at each site in the genome from bam alignment files. As these mutations were absent at the beginning of the experiment, the frequency of these mutations at the end of the experiment also represents their allele frequency change. We used the Popoolation2 implementation to calculate nucleotide diversity.

To quantify how temperature and host regimes facilitate or constrain rates of parasite evolution, we calculated genomic divergence from the ancestor in each experimental population and treatment, by summing the frequency of allele frequency change across all loci. Additionally, we calculated a matrix of Euclidian distances across treatments for allele frequency change at all loci. Euclidian distance from the ancestor was calculated as the distance from zero for each locus, alongside each within‐treatment pairwise distance.

To investigate signals of adaptive evolution over the course of the experiment, we took two approaches. One approach was to identify mutations present across multiple populations within each treatment to identify those involved in convergent, repeatable genomic evolutionary responses. To do so, we leveraged the independent populations within each experimental treatment to quantify the mean and standard error of allele frequency change and calculated the corresponding test statistic and significance based on a *p*‐value cutoff of alpha = 0.05. The second approach was to identify *de novo* mutations that had dramatically increased in frequency over the course of the experiment. For the 810 *de novo* mutations present across at least one population, we quantified the number of populations it was present in, which temperature regime it evolved under, which host regime it evolved under, and its location in the genome relative to the reference. For all following analyses, we combined these approaches by only including variants that either displayed significant repeatable evolution within an experimental treatment, or failing that, had minor allele frequencies (MAF) of at least 10%. Doing so meant we could focus on loci most likely to be under selection, without omitting potentially important rare mutations.

To test whether genome‐wide allele frequency change and diversity (in the absence of recombination and migration, due to only mutation, selection and drift) corresponded to experimental treatment, we performed a multiple linear regression to test whether host, temperature and host × temperature, and presence across experimental populations explained each independent variable. We expected that mutations that appeared across multiple populations would be those most likely representing a convergent response to selection. If so, we might expect that such mutations also show the most allele frequency change over the course of the experiment. We performed these multiple regression analyses using the lm function and a type 3 Anova in R (Fox and Weisberg 2019). As the number of populations varied across infection treatments we normalised population number as a predictor such that it would be comparable across treatments. We then extracted the least squares means from the multiple regression analysis, using the function lsmip in R (Lenth 2022). This analysis allowed us to pull out the mean temperature and host effect on allele frequency change/diversity, while accounting for the modelled interaction effect.

### 2.10 Statistical Analysis of Phenotypic Data

All statistical analyses were performed in R [v. 4.2.1 (R Core Team 2022)]. Data wrangling and figures were produced using the ‘Tidyverse’ (Wickham et al. 2019), ‘cowplot’ (Wilke 2019), ‘ggpubr’ (Kassambara 2023a). Descriptions of model structure and error distributions for statistical models are shown in Tables S10 and S11. Our unit of biological replication was each independent, evolving population. So, where appropriate, ‘population’ was fitted as a random effect to account for the 3–5 technical replicates per population/assay temperature combination. Mixed models were built using the packages ‘lme4’ (Bates et al. 2015) and ‘glmmTMB’ (Brooks et al. 2017), and non‐parametric tests used the ‘Rstatix’ package (Kassambara 2023b). Models were assessed using the ‘DHARMa’ package (Hartig 2022). Significance of fixed effects was assessed with Analysis of Variance (ANOVA, Type III; ‘car’ package (Fox and Weisberg 2019)). Post hoc pairwise tests were performed using the ‘emmeans’ package (Lenth 2022).

To analyse the relationship between virulence and within‐host burden we first predicted average trait values for host mortality and CFUs. For host‐evolved populations, we predicted for each level of the random effect, that is, we extracted the mean predicted values for each population (*n* = 6 for each evolution temperature treatment). For environmental control treatments, we predicted the mean treatment‐level main effect. In this analysis we were interested in evolutionary change that is specific to association with a host. We therefore calculated the relative host effect of both traits for each host population by subtracting the predicted mean of their respective environmental control. For each assay temperature both traits were then centred and scaled prior to analysis with Multivariate Analysis of Variance (MANOVA; ‘car’ package (Fox and Weisberg 2019)). Kendall's Rank correlation was used to assess the within‐treatment correlations.

## 3 Results

Disease outcomes for *C. elegans* and the ancestral *L. musarum* isolate varied across the parasite's environmental temperature range. Parasite‐induced host mortality—a proxy for virulence—peaked during infections at 25°C, the average warm environmental temperature at the parasite's site of collection (Figure 1C). At 20°C, towards the hosts thermal optima, *L. musarum* is less virulent to the *C. elegans* host (Figure 1C). Virulence also declined during infections at hot temperatures closest to the parasite's optimum (30°C, Figure 1C) (Will et al. 2025), despite a trend for increased in vitro growth and replication within hosts (parasite load, e.g., (de Roode et al. 2008)) (Figure S1; Kruskal–Wallis Test *χ* ^2^ = 5.956, df = 2, *p* = 0.051; Dunn post hoc test 25°C–30°C, *z* = 2.385, *p* = 0.017, adj. *p* = 0.051). Following a short experimental heatwave at 35°C uninfected *C. elegans* experience mortality which is amplified by infection (Figure 1D; ANOVA, *F*_3,12_ = 342.2, *p* = < 0.001; infected vs. uninfected Tukey's post hoc test *t* = 15.202, *p* = < 0.001), despite the parasite simultaneously experiencing reduced cell viability relative to ambient temperatures (Figure S3; Kruskal–Wallis Test *χ* ^2^ = 14.552, df = 1, *p* = < 0.001).

### 3.1 Virulence of Evolved Populations

We first tested whether there was a difference in the virulence of host‐evolved parasites compared to the ancestor. Overall, within‐host evolution at 25°C and 30°C selected for the highest levels of virulence during infections at 25°C, an increase of 4% and 3%, respectively (25°C: EMM = 4.054, 95% CI [0.968, 7.140], *t* = 2.646, *p* = 0.011). When evolved at cooler temperatures favourable to the host (20°C), parasites maintained ancestral levels of virulence during subsequent infections at both cool and warm temperatures (Table S1). These 20°C parasites were also generally less virulent compared to the ancestor during assay infections at 30°C (EMM = −6.881, 95% CI [−0.966, −3.795], *t* = −4.491, *p* = < 0.001). For parasite populations evolved at 30°C, virulence was similar to the ancestor during infections at the same hot temperature (EMM = −1.989, 95% CI [−5.075, 1.097], *t* = −1.298, *p* = 0.201). Yet, these parasites were more virulent compared to the ancestor during assay infections at a lower 25°C (EMM = 3.328, 95% CI [0.243, 6.414], *t* = 2.173, *p* = 0.035).

We then assessed changes in virulence across infection treatments, evolution temperature and assay temperature (Figure 2B, Tables S2 and S3). Virulence depended on a two‐way interaction between the presence/absence of hosts and evolution temperature (*χ* ^2^ = 17.901, df = 2, *p* = < 0.001). Across evolution treatments, virulence varied depending on the assay temperature (*χ* ^2^ = 745.6287, df = 2, *p* = < 0.001). There was no evidence for higher‐order interactions between assay temperature and either experimental evolution treatment (Table S2). Parasites evolved within hosts at 25°C were on average 20% deadlier than environmental controls during infections across the full constant temperature range (Host/Environmental back‐transformed estimate = 0.201, SE = 0.028, *z* = 7.108, *p* = < 0.001). Virulence was also lower in environmental controls relative to host‐evolved parasites when passaged at 20°C (estimate = 0.154, SE = 0.028, *z* = 5.556, *p* = < 0.001). In contrast, for 30°C environmental control parasite populations, virulence was maintained at levels comparable to host‐evolved populations across the full constant temperature range (Figure 2B; Table S3).

Parasites passaged through regular heatwave conditions saw no change in virulence across infection treatments, or relative to the ancestor, during heatwave or constant temperature assays (Figure 2B and Figure S4A; Tables S4–S6). However, host‐evolved parasites passaged at 20°C, 25°C and 30°C expressed higher virulence relative to their respective environmental controls (Table S5) and the ancestor (Table S6) during subsequent heatwaves (Figure 2B and Figure S4B).

### 3.2 Virulence‐Transmission Trade‐Off

For each parasite population evolved at constant temperatures we also measured within‐host colony forming units (CFUs) as a proxy for replication/infection burden. Overall, within‐host levels of evolved replication were highest at 30°C (Figure S5). We used this measure of replication to explore how temperature had influenced the evolutionary relationship between burden/replication and host mortality. We found that virulence and replication evolved along different trajectories across temperatures (Figure 2C and Figure S6) (Table S7; MANOVA, interaction between evolution and assay temperature for host‐evolved populations: Pillai statistic = 0.549, *F*_8,90_ = 4.256, *p* = < 0.001). Within‐host evolution during warming led to a positive correlation between virulence and replication (Figure 2C; 25°C: Kendall's rank correlation *τ* = 0.966, *z* = 2.678, *p*‐value = 0.007, *n* = 6). In contrast, passage at cooler temperatures towards the host's optimum and hot temperatures where parasite replication peaks (Figure S5), broke down this relationship (Figure 2C; 20°C: Kendall's rank correlation *τ* = −0.276, *z* = −0.765, *p*‐value = 0.444, *n* = 6; 30°C: Kendall's rank correlation *τ* = 0.333, *z* = 0.939, *p*‐value = 0.348, *n* = 6).

**FIGURE 3:**
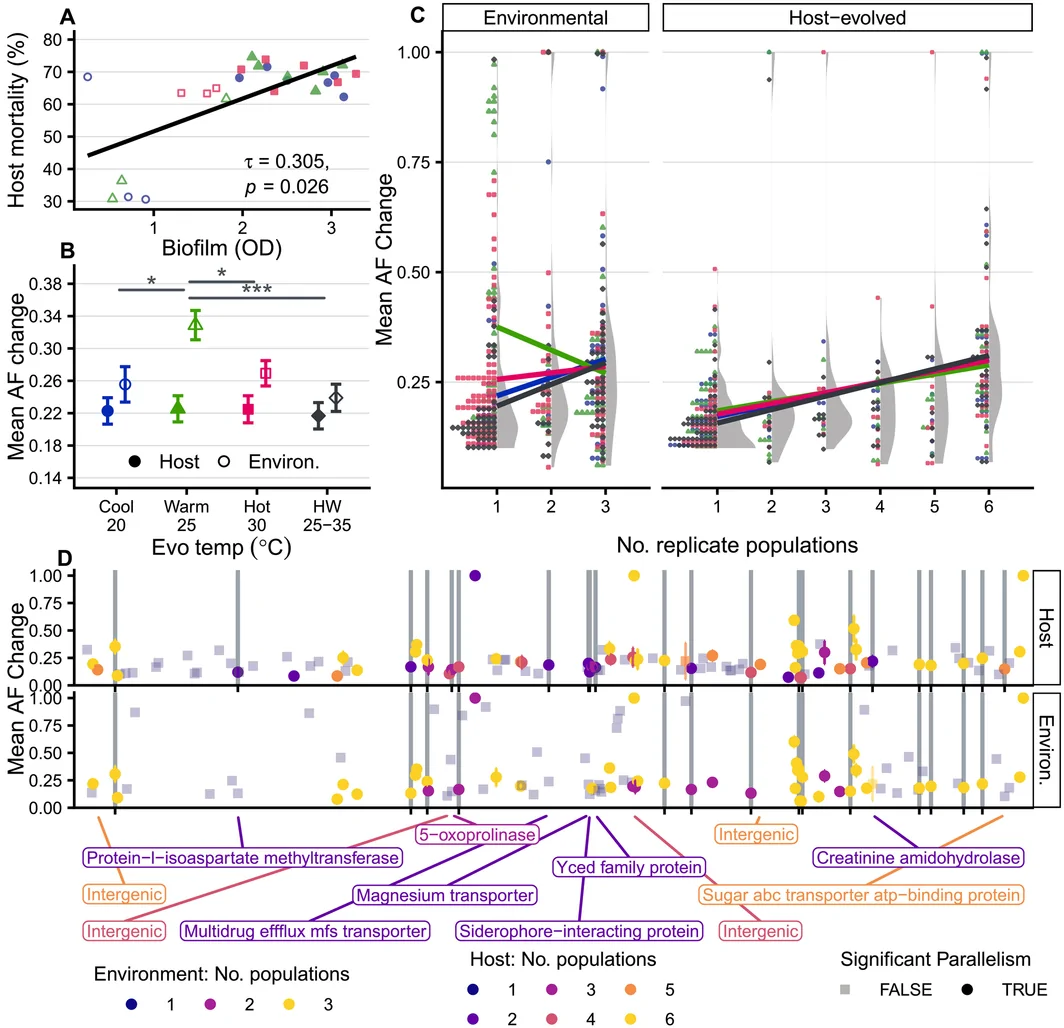
Temperature and hosts drive parasite molecular evolution. (A) Relationship between biofilm formation [optical density (OD_575_)] and host mortality across evolved populations. Points show the mean trait value for each parasite population measured at 25°C; colours and shapes match panel B. Text shows the significant Kendall's rank correlation coefficient across all populations (*n* = 27). (B) Predicted mean (± SE) minor allele frequency (MAF) of *de novo* variants across host‐evolved (Host) and environmental control (Environ.) parasite populations from each evolution temperature (variants displaying FDR corrected significant parallel change across populations or unique variants with MAF > 0.1). Asterisks show significance of pairwise comparisons between environmental parasites passaged at 25°C and each other temperature (pairwise *t*‐test's 25°C–20°C: T = −2.689, *p* = 0.021 *, 25°C–30°C: T = −2.637, *p* = 0.024 * and 25°C–HW: T = −3.776, *p* = < 0.001 ***). (C) Mean MAF as a function of the number of replicate populations in which a SNP arose in parallel. Colours and symbols match panel B. (D) Mean MAF by genomic position for host‐evolved and environmental controls passaged at 25°C. Colours show the level of parallelism across populations (note that host‐present treatments had six populations and environmental controls had three), transparent square points show variants that did not display significant parallel evolution. Grey bars show parallel variants within genes (synonymous and non‐synonymous). Labels below highlight the predicted functions of genes containing variants that were unique to host‐evolved populations.

The absence of a universal relationship between replication and virulence suggested that traits other than replication were likely involved in the context‐dependent evolutionary shifts in virulence. We measured in vitro growth and biofilm formation of evolved parasite populations. Overall, in vitro growth rates were highest at 30°C (Figure S7; *t* = 2.213, *p* = 0.0352), while environmental control populations evolved at 25°C experienced a reduction in lag time (Figure S8; *t* = 2.421, *p* = 0.0222). We found a positive correlation between population in vitro lag time and host mortality for parasites evolved at intermediate 25°C and 30°C (Figure S8; Pearson's correlation *R* = 0.55, *p* = 0.019). We also found that the environmental parasites displayed the lowest rates of biofilm formation, while all host‐evolved parasites evolved increased biofilm formation ability (Figure S9; ANOVA, *F*_5,21_ = 14.851, *p* = < 0.001; 20°C host/environment post hoc: *t* = −6.518, *p* = < 0.001; 25°C host/environment: *t* = −4.906, *p* < 0.001; 30°C host/environment post hoc: *t* = −3.257, *p* = 0.0382). The divergence in both virulence and biofilm formation between infection treatments (Figure 2B and Figure S9) led to a positive correlation across all populations (Figure 3A; Kendall's correlation *τ* = 0.305, *p* = 0.026).

### 3.3 Population Genetics of Host‐ and Thermal‐Adaptation

Using pooled whole genome sequencing, we quantified the evolution and putative signals of selection on *de novo* variants arising over ten host generations (Figure 2A). Across treatments, there were approximately 70 *de novo* variants per population with 4–8 SNPs reaching allele frequencies > 50% (Figure S10A; Table S8). For host‐evolved parasite populations, ~22% of variants were unique to each temperature treatment and 10% were shared across all treatments (Figure S11). Within coding regions, 70% of variants were non‐synonymous substitutions (Figure S10B) indicating potential targets of selection, many within genes linked to biofilm formation, virulence, metabolism and abiotic stress responses (Figures S12 and S13), suggesting a polygenic basis to shifts in virulence.

Parasite molecular evolution across the genome was on average lower, but more predictable, within hosts compared to those in the environment. When looking at common mutations (minor allele frequency (MAF) > 0.1), within‐host evolution generated the lowest genome‐wide diversity across the full thermal range (Figure 3B; Table S9; ANOVA, infection treatment: *F*_1,822_ = 24.067, *p* = < 0.001). The degree of population divergence, measured as the sum of allele frequency change (ANOVA, infection treatment: *F*_1,28_ = 12.937, *p* = 0.001); Euclidean genetic distance from the ancestor (ANOVA, *F*_1,28_ = 8.633, *p* = 0.007; Tukey post hoc test *t* = 2.938, *p* = 0.007); and within‐treatment diversification (*t* = −8.982, *p* = < 0.001) were also reduced across infection treatments (Figure S14). We investigated the extent to which the same *de novo* mutations evolved independently across populations in response to host or temperature‐mediated selection, as evidence for adaptive evolution (Bolnick et al. 2018). Evolving within hosts drove the most molecular parallelism (≥ 2 populations; Figure 3C; Table S9; host/environment pairwise *t*‐test = −3.41, *p* = < 0.001), with evidence for variants in genes linked to infection repeatedly arising, persisting and increasing in frequency across populations, consistent with selection (Figure 3D).

The impact of temperature on the genetic architecture of parasite adaptation was most distinct in the environmental populations. Intermediate warm temperatures (25°C) facilitated the fastest rates of evolution, with the largest changes in mean allele frequencies genome‐wide (Figure 3B; Table S9). Environmental populations evolving in warm conditions also showed relaxed evolutionary constraint, with higher rates of allele frequency change for non‐parallel (unique to a single population) mutations (Figure 3C; linear model, β = −0.045, df = 822, *t* = −2.753, *p* = 0.006). In contrast, parasite populations in cool and heatwave conditions experienced the lowest average allele frequency changes (Figure 3B) and nucleotide diversity (ANOVA, heatwave parameter estimate, *t* = −2.681, *p* = 0.012; Figures S14 and S15).

## 4 Discussion

Changing environmental temperatures are expected to have far‐reaching impacts on infectious disease ecological dynamics (Ferguson and Adamo 2023; Li et al. 2024; Mahon et al. 2024; Greenrod et al. 2025). Yet, how parasites may evolve in response to shifting host and thermal environments remains unclear. We predicted that warm and hot temperatures favourable to the parasite would promote higher virulence in the cool adapted host. We saw that the highest level of evolved virulence occurred following within‐host evolution at either warm (25°C) or hot (30°C) temperatures, when subsequent infections occurred at 25°C. Infection temperatures that spanned the thermal mismatch therefore resulted in distinct evolutionary outcomes for parasite virulence. Our findings suggest that during within‐host evolution, virulence may be most likely to increase at intermediate temperatures between host and parasite thermal optima. The threat to host populations from emerging and sympatric parasites alongside unseasonably warming temperatures (Carlson et al. 2017; Cohen et al. 2020) could be amplified by rapid parasite evolution.

The virulence‐transmission trade‐off predicts virulence and replication will positively correlate in environments optimal for host exploitation (Anderson and May 1982; Frank 1996; Alizon et al. 2009). These parasite traits can thus be decoupled in contexts where host exploitation is suboptimal (Bull and Ebert 2008; Tardy et al. 2019; Alizon and Sofonea 2021; Ekroth et al. 2021; Silva and King 2025). We therefore expected the relationship between virulence evolution and within‐host replication to be strongest under hot conditions (30°C) where parasite growth is highest. However, we found that virulence and replication evolved along different trajectories across temperatures. Our results suggest that temperature can cause these parasite traits to evolve along independent trajectories during thermal mismatches. Rather than temperatures optimal for parasite growth resulting in a positive association between within‐host growth and virulence (Acevedo et al. 2019), we found this relationship emerged at intermediate temperatures between the host and parasite thermal optima. The predictability of parasite virulence evolution will be context dependent (Hector et al. 2023; Silva and King 2025), potentially deviating from expected trajectories when host and parasite thermal niches do not align.

Hot temperatures—beyond the host's thermal maxima—drove an evolved increase in virulence which was apparent at lower temperatures. At sympatric temperatures, parasite virulence was similar to the ancestor and environmental controls following passage at 30°C. Yet, when returned to warm conditions, these hot‐evolved parasites were more virulent compared to the ancestor. Higher virulence is predicted as an evolutionary outcome when hosts are tolerant to infection (Little et al. 2010; Smith and Ashby 2023). In *C. elegans* , as in most eukaryotes, heat triggers multiple stress response and immune systems, such as the heat‐shock and unfolded protein responses, which can confer either resistance or tolerance to infection (Singh and Aballay 2006; Richardson et al. 2010; Kumsta et al. 2017; Ferguson and Adamo 2023). We saw that on average, nematodes could better withstand all parasite populations during constant hot conditions while maintaining relatively high parasite loads. So, hotter hosts are not necessarily sicker. We speculate that physiological responses to heat stress which increase host tolerance to infection could encourage parasite variants which are deadlier when temperatures cool and tolerance subsides. This evolutionary process may play a role in mass mortality events and seasonal disease outbreaks from bacterial infections following unusually hot weather (Fey et al. 2015).

More frequent heatwaves are accompanying the warming in average temperatures globally (Fischer et al. 2021). The peak soil temperatures in Cabo Verde (35°C) are increasingly also occurring in temperate regions (Christidis et al. 2020). Infection is predicted to be a major driver of mortality during extreme thermal events (Hector et al. 2021). At an ecological level, we see that infected *C. elegans* were more likely to experience mortality compared to healthy individuals during an experimental heatwave. However, despite the virulence of infection, *L. musarum* also experienced reduced viability following the heatwaves. These findings reveal how temperature shifts can alter the nature of the virulence‐replication relationship, with heatwaves driving virulent infections despite fitness costs for the parasite (Hector et al. 2023; Ragonese et al. 2024). Across evolutionary time, however, parasite populations passaged under regular heatwaves maintained ancestral levels of virulence, regardless of evolution in hosts or the environment. The low rates of parallel genomic evolution for parasites passaged through heatwaves indicate strong selection on a limited range of beneficial variants (Orr 2005; Barghi et al. 2020). Simultaneously, genomic variation and diversification could have been limited by both purifying selection and genetic drift due to high parasite mortality.

Many bacterial parasites can proliferate in the environment. Understanding the impact of temperature on evolutionary trajectories during environmental residency is crucial to forecasts of infection outcomes upon host contact (Pandey et al. 2022). We introduced parasite populations evolved in vitro across temperatures (i.e., environmental controls) to host populations. We found that virulence was reduced following environmental passage at cool and warm temperatures. A positive relationship between in vitro lag time and virulence points towards a functional trade‐off between environmental growth and virulence. The reduction of virulence in the environmental parasites also coincided with the lowest rates of biofilm formation. These results support the prediction that periods of environmental residency can select against costly virulence‐related traits (Mikonranta et al. 2012; Maury et al. 2019; Pandey et al. 2022), which may restrain opportunistic parasites from reaching their maximum virulence at ambient temperatures due to antagonistic selection pressures within and outside of the host (Mideo et al. 2008). In contrast, at hot and heatwave temperatures the virulence of environmental parasites was maintained. Consequently, when returned to warm or cool temperatures, hot‐evolved parasites remained highly virulent irrespective of their in vitro or in vivo evolutionary history. Highly deadly variants residing in the environment may thus be conserved as temperatures continue to escalate.

The signatures of temperature‐mediated selection on parasite genomes differed depending on whether the parasite was evolving in a host or in the environment. Parasite molecular evolution across the genome was on average lower, but more predictable, within hosts compared to those in the environment. We investigated the extent to which the same *de novo* mutations evolved independently across populations in response to host or temperature‐mediated selection, as evidence for adaptive evolution (Bolnick et al. 2018). Evolving within hosts drove the most molecular parallelism, with evidence for variants in genes linked to infection repeatedly arising, persisting and increasing in frequency across populations, consistent with selection. Higher levels of genetic parallelism are likely to arise when the genetic architecture is encoded by few large effect alleles (Barghi et al. 2020) or the adaptive mutational target size is low (Orr 2005), such as in extreme environments (Behringer et al. 2024) and in parasites under strong host‐mediated selection (Marvig et al. 2015; Vogwill et al. 2016). Here, our findings are consistent with host‐mediated selection constraining the size of the adaptive landscape, dominating population genomic changes relative to temperature. The lack of a genome‐wide signal of temperature for host‐evolved parasites suggests that phenotypic changes in virulence are likely the result of a few large effect loci or polygenic genetic architecture with distinct (non‐parallel) changes across replicate populations, rather than parallel polygenic adaptation at each temperature (Barghi et al. 2019, 2020; Whiting et al. 2024). While functional validation of specific candidate genes identified here may contribute to current and future genomic surveillance efforts during climate‐driven epidemics (Espinoza et al. 2023), polygenicity in within‐host virulence evolution may pose a challenge to these efforts.

The impact of temperature on the genetic architecture of parasite adaptation was most distinct in the environmental populations. We hypothesised that in the absence of host‐mediated selection, constant hot conditions would promote rapid molecular evolution due to higher rates of replication and mutation, whereas strong selection at extreme temperatures would constrain evolutionary rates (Chu et al. 2018; Zhang et al. 2018; Liu et al. 2023). Instead, warm temperatures (25°C) facilitated the fastest rates of evolution, with the largest changes in mean allele frequencies genome‐wide. These evolutionary rates do not simply align with the evolution of replication rates across temperatures (Berger et al. 2021)—both in vitro and within‐host replication were highest at the hottest temperature (30°C). Environmental populations evolving in warm conditions also showed relaxed evolutionary constraint, with higher rates of allele frequency change for non‐parallel (unique to a single population) mutations. While evolution driven by many population‐specific SNPs may be the result of highly polygenic adaptation (Barghi et al. 2019, 2020; Whiting et al. 2024), it can also indicate weak selection or an increased contribution of genetic drift. In contrast, parasite populations in cool and heatwave conditions experienced the lowest average allele frequency changes and nucleotide diversity. Analogous to all host‐adapted populations, SNPs that did spread at extreme temperatures in the environment were more often parallel across populations, consistent with a subset of alleles with strong phenotypic effect size involved in convergent adaptation. Our results suggest parasite evolution may become more predictable, and thus manageable, during infectious disease outbreaks as the climate continues to warm.

The eco‐evolutionary dynamics of host–parasite interactions under environmental change are inherently complex (Pfenning‐Butterworth et al. 2024; Poulin et al. 2024). Making broad predictions across taxa and environments is challenging. Our experiment in a model system provides a proof of principle that temperature can drastically shape the virulence and genetic architecture of adaptation for bacterial parasites, growing in a new host or in the environment. We found that warm temperatures typical of the natural environment for the tropical parasite maximised the capacity for a virulence boost during within‐host evolution. The host–parasite thermal mismatch further imposed selection on parasites with constant heat favouring or maintaining virulent variants that are only revealed at cooler or extreme temperatures, which we hypothesise could result from shifting temperature‐mediated host tolerance. The increasing severity of warming and disease outbreaks are already placing many species at risk (Claar and Wood 2020; Hector et al. 2021; Li et al. 2024). The rapid evolution of (potentially latent) virulence will intensify these threats. Yet, any reduction in the pace of adaptation at more extreme temperatures could increase the window of opportunity for the development of effective clinical interventions to emerging infectious diseases. Further investigations should assess whether the timing of mass mortality events in animal populations is similarly linked with extreme temperature patterns. Experimental and field studies across a diverse range of hosts and parasites would aid in identifying the risk faced by host species in warming environments, and whether predictable genomic architectures underlie parasite adaptation. Direct comparisons of parasite evolutionary outcomes during adaptation to thermally matched versus mismatched hosts (e.g., *C. tropicalis* vs. *C. elegans* ) would provide further insight into the interactions between host and parasite responses to changing thermal conditions.

The short‐term evolutionary timescales explored here are predicted to be particularly relevant for determining the establishment and severity of emerging disease outbreaks (Antia et al. 2003; Hawley et al. 2013). This timescale is also relevant considering many climate‐related disease outbreaks are likely to be seasonal (Altizer et al. 2006) or in host populations that undergo ‘boom and bust’ population dynamics (Bubrig and Gibson 2025). Whether warming also reshapes longer‐term evolutionary strategies and the coevolutionary dynamics between thermally mismatched hosts and emerging parasites remains an open question. Going forward, we should also consider climate data alongside geographic range overlap (Carlson et al. 2022), host phylogenetic relatedness (Shaw et al. 2020; French et al. 2023), and environmental residency (Pandey et al. 2022) in epidemiological (Pfenning‐Butterworth et al. 2024) and evolutionary predictions of outbreaks. This focus could be particularly informative for getting ahead of virulent variants emerging at the edge of expanding ranges or within existing host habitats where temperatures are becoming extreme (Carlson et al. 2017; Price et al. 2019; Ryan et al. 2019; Cohen et al. 2020).

## Author Contributions

**James C. Forward:** investigation, writing – review and editing, methodology. **Tobias E. Hector:** conceptualization, methodology, data curation, investigation, formal analysis, visualization, writing – original draft, writing – review and editing, funding acquisition. **Kim L. Hoang:** investigation, formal analysis, writing – review and editing. **Serena Johnson:** investigation, writing – review and editing. **Kayla C. King:** conceptualization, funding acquisition, project administration, writing – original draft, writing – review and editing, methodology. **Julia M. Kreiner:** formal analysis, data curation, writing – review and editing, visualization, investigation. **Jingdi Li:** investigation, writing – review and editing. **Emily J. Stevens:** investigation, data curation, writing – review and editing.

## Funding

Philip Leverhulme Prize (K.C.K.). NERC Environmental Omics Facility grant (no. NEOF1368) (K.C.K., T.E.H.). NERC Research Project Grant (NE/X000540/1) (K.C.K.). NSERC Canada Excellence Research Chair (K.C.K.).

## Conflicts of Interest

The authors declare no conflicts of interest.

## Supporting information


**Figure S1:** In vitro growth metrics and within‐host burden for the ancestral parasite clone across temperature. (A) Average optical density (OD600) measurements across time for *L. musarum* samples grown at either 25°C or 30°C. (B) Average lag time at each temperature. (C) Average maximum growth rate. Metrics were calculated using the gcplyr R package (79) (see methods, *n* = 3 per temperature). (D) Colony Forming Units (CFU)—a measure of within‐host bacterial load—of ancestral parasite isolate across three constant environmental temperatures (±SE). While the effect of temperature here was marginally non‐significant (Kruskal–Wallis Test: statistic = 5.956, df = 2, *p*‐value = 0.051), there was a trend towards increased numbers of within‐host bacterial cells at 30°C (Dunn Test 25°C–30°C: statistic = 2.385, *p*‐value = 0.017, adj. *p*‐value = 0.0512, *n* = 3 per temperature).
**Figure S2:** Phylogeny and collection locations for a subset of European *C. elegans* wild‐type isolates. Tip label colours and map points represent the genomic distance from the domesticated N2 strain. Data from: Crombie et al. (2023).
**Figure S3:** Viability of *L. musarum* following an experimental heatwave. (A) In vivo cell viability (±SE). Colony forming units within host *C. elegans* after exposures at either 25°C or 35°C heatwave. The trend for reduced CFU following heatwave was not significant (Kruskal–Wallis Test: statistic = 1.191, df = 1, *p*‐value = 0.275, *n* = 3 per temperature). (B) In vitro cell viability (±SE). Twelve overnight culture of *L. musarum* grown from individual colonies were distributed evenly across three 96‐well plates and maintained at 25°C or exposed to a 3‐h experimental heatwave at 35°C. Following the heatwave, all replicates were spotted on LB agar and incubated at 25°C for 48 h, after which the number of colonies were counted. There were significant fewer viable cells following heatwave (Kruskal–Wallis Test: statistic = 14.552, df = 1, *p*‐value = < 0.001, *n* = 12 per temperature).
**Figure S4:** Mortality of *C. elegans* exposed to evolved *L. musarum* populations (or the ancestral isolate). (A) Mortality was measured for host‐evolved (Host) and environmental control (Enviro.) parasite populations evolved under heatwave (and the ancestral isolate) during assays at constant 25°C or 30°C (facets). (B) Mortality of host‐evolved and environmental control parasite populations evolved at 20°C, 25°C or 30°C measured during a heatwave assay.
**Figure S5:** Within‐host colony forming units (CFUs) depend on evolution treatment and assay temperature. Within‐host CFUs from nematode hosts exposed to the ancestral isolate (ANC), hostevolved, or environmental controls (enviro) populations at either 20°C, 25°C or 30°C. Parasite CFUs of all populations were assayed at each constant temperature (facets). Points are the average of three technical replicates.
**Figure S6:** Raw data (scaled, and centred on ancestor) for colony formatting units (within‐host burden) and virulence (mortality) of *L. musarum* populations host‐evolved at 20°C, 25°C or 30°C. Traits were measured for populations from all treatments at each temperature in subsequent assays (facets). Coloured text shows Pearson's correlation coefficients for each treatment and temperature (20°C = blue, 25°C = green, 30°C = pink). As with the data shown in Main Figure 2C, there is only a positive relationship between within‐host burden and host mortality for parasite populations passaged at warm 25°C (*n* = 6 per treatment combination).
**Figure S7:** In vitro growth rates for parasite populations at two test temperatures (25°C and 30°C). Shown are the calculated maximum growth rates (μmax) for parasite populations or the ancestral isolate during in vitro growth in 96‐well plates at either 25°C or 30°C (facets). Both host‐evolved (Host) and environmental controls (Environ.) are shown.
**Figure S8:** The relationship between mean in vitro lag time and mean virulence (host mortality) for each population passaged at 25°C (green) and 30°C (pink) host‐evolved (circles) or environmental controls (triangles), at (A) 25°C, or (B) 30°C assay temperatures. Inset text shows Pearson's correlation coefficients (*n* = 18).
**Figure S9:** Increased biofilm formation in host‐evolved parasite populations. Average biofilm formation (measured as optical density 600 nm) for parasite populations and the ancestral isolate. Biofilm formation was measured for populations grown at 25°C. ANC, ancestral isolate; Environ., environmental controls; Host, host‐evolved treatments. Each point is the mean of six technical replicates.
**Figure S10:** Single nucleotide polymorphism (SNP) counts for parasite populations from each treatment. (A) Counts of variants (genome‐wide) for each population from pools of 40 sequenced clones. The x‐axis shows each independent population. (B) Average SNP count (±SE) per treatment for different mutation type. Transparent points show counts for each independent population.
**Figure S11:** Most single nucleotide polymorphisms (SNPs) are either shared across all treatments or unique to a single treatment. Shown are the number of SNPs (genome‐wide) that occurred in within and across different combinations of treatments for host‐evolved parasite populations.
**Figure S12:** Mean allele frequency change across host‐evolved populations from each evolution temperature. Shown are SNPs along the genome. Colour represents the number of populations a SNP arose in parallel, non‐translucent points showed significantly higher allele frequencies than would be expected at random in parallel SNPs. Grey bars show the position of non‐synonymous genic SNPs which showed significant allele frequency change in parallel across multiple populations (with the corresponding putative functions below).
**Figure S13:** Putative functions and number of genes potentially under selection. For each evolution treatment, shown are the average SNP counts (colours) and average proportion relative to all SNPs in that treatment (numbers) for each putative functional category. Only SNPs within genes are shown. The data was further filtered to only include SNPs with minor allele frequencies > 10% or variants which showed significant parallel evolution across populations within treatment.
**Figure S14:** Genetic distance and divergence of host‐evolved parasite populations across temperatures. (A) Genetic divergence as the sum of allele frequency change for populations in each treatment, (B) Euclidean distance of each population to the ancestor from all SNPs (MAF > 0.1). (C) Euclidean distance between populations within each evolution treatment, showing within treatment diversification. Large black points show the treatment mean (± standard error), coloured points show the population means (A and B) or mean for each specific population‐population comparison (C).
**Figure S15:** Nucleotide diversity for host‐evolved parasite populations declines at the hottest temperature treatments. Per gene nucleotide diversity, predicted using the PoPoolation2 software (87) following the standard workflow (https://sourceforge.net/p/popoolation2/wiki/Main/). Data shows the mean (± SE) of the average genome‐wide nucleotide diversity of populations within each treatment.
**Table S1:** Host mortality following exposure to host‐evolved populations passaged at constant temperatures relative to the ancestor. Parameter estimates from a linear mixed effect model testing the effects of evolution temperature (20°C, 25°C and 30°C) and assay temperature (20°C, 25°C and 30°C) on mortality for host‐evolved parasite populations relative to the average ancestral value. Host‐evolved population values were centred on the average ancestral value at each assay temperature and modelled using a zero‐intercept linear mixed effect model. The difference from zero of estimated marginal means was assessed using *t*‐tests.
**Table S2:** Virulence (host mortality) of parasite populations passaged at constant temperatures during assays across the three constant temperatures (20°C, 25°C and 30°C). Shown are the significance of fixed effect estimated from a model of host mortality, predicted by evolution temperature treatment (levels = ‘20’, ‘25’ and ‘30°C’), infection treatment (levels = ‘hostevolved’ and ‘environmental control’), assay temperature (levels = ‘20’, ‘25’ and ‘30°C’), and all higher order interactions. The data were modelled using a generalised linear mixed effect model (GLMM) with a Beta‐binomial (log link) error distribution using the ‘glmmTMB’ package. Fixed effects were estimated with Analysis of variance (ANOVA, type III), using the ‘car’ package.
**Table S3:** Pairwise comparisons between host‐evolved and environmental control populations at each level of evolution temperature, averaged across assay temperature due to the lack of higher order interactions. Estimated from the GLMM reported in Table S2. Contrast were calculated on estimates back‐transformed to the original response scale and so estimates represent proportional differences. Bonferroni corrected *p*‐values are shown.
**Table S4:** Virulence (host mortality) of parasite populations passaged at 25°C, 30°C, or under the heatwave temperatures during simulated heatwave (25°C–35°C) assays. Shown are the significance of fixed effect estimated from a model of host mortality, predicted by evolution temperature treatment (levels = ‘25’, ‘30°C’ and Heatwave), infection treatment (levels = hostevolved and environmental control), and their interaction. The data were modelled using a generalised linear mixed effect model (GLMM) with a Beta‐binomial (log link) error distribution using the ‘glmmTMB’ package. Fixed effects were estimated with Analysis of variance (ANOVA, type III), using the ‘car’ package.
**Table S5:** Pairwise comparisons between host‐evolved and environmental control populations at each level of evolution temperature during a heatwave assay. Estimated from the GLMM reported in Table S5.
**Table S6:** Parameter estimates from a linear mixed effect model testing the effects of evolution temperature (25°C, 30°C and heatwave) on mortality under heatwave conditions for hostevolved parasite populations relative to the average ancestral value. Host‐evolved population values were centred on the average ancestral value and modelled using a zero‐intercept linear mixed effect model. The difference of estimated marginal means from zero was assessed using *t*‐tests.
**Table S7:** Significance of fixed effects from a MANOVA testing the effects of evolution temperature (20°C, 25°C, 30°C) and assay temperature (20°C, 25°C, 30°C) on the relationship between relative host mortality and relative within‐host colony forming units (CFU), for host‐evolved parasite populations. Relative trait values were produced by subtracting the average environmental control's value from the matching evolution temperature treatment. This relative measure allows the partitioning of changes to phenotypic traits that were specific to within‐host processes.
**Table S8:** Counts of genome‐wide de novo variants at different levels of minor allele frequency (MAF). Average counts are shown for each experimental evolution treatment, with the standard deviation (SD).
**Table S9:** ANOVA (Type III) of a fully parameterised model showing the significance of fixed effects for the effect of evolution temperature (20°C, 25°C, 30°C and Heatwave), infection treatment (host‐evolved and environmental controls), and level of parallel evolution (No. populations) on mean allele frequency of variants within evolution treatments. The number of within‐treatment populations each de novo variant arose in (No. populations/Scaled reps) was scaled to between zero and one, to account for the differing number of populations across treatments.
**Table S10:** Details of statistical models analysing host mortality or colony forming units (CFU) phenotype data. Each model assessed the effects of experimental ‘Evolution temperature’ treatment, ‘Infection treatment’, ‘Assay temperature’ (temperature at which traits were measured at the end of the experimental evolution), or combinations thereof, as fixed effects. Generalised Linear Mixed Models were fitted using the ‘glmmTMB’ package (96), and Linear Mixed Models using ‘lme4’ package (95). Fixed and random effect structures were determined by experimental design (maximal models) and model fit diagnostics were assessed using the ‘DHARMa’ package (98). Temperatures include 20°C, 25°C, 30°C and the experimental heatwave (HW, see methods). Model structures are shown using R syntax (additive effect = ‘+’, interaction = ‘*’, nested random effect = ‘/’). Where appropriate the significance of fixed effects was tested using Analysis of Variance (ANOVA type III) using the ‘car’ package (89).
**Table S11:** Details of statistical models analysing colony forming units (CFU) phenotype data. Each model assessed the impact of experimental ‘Evolution temperature’ treatment, ‘Infection treatment’, ‘Assay temperature’ (temperature at which traits were measured at the end of the experimental evolution) or combinations thereof, as fixed effects. Generalised Linear Mixed Models were fitted using the ‘glmmTMB’ package (96), and Linear Mixed Models using ‘lme4’ package (95). Fixed and random effect structures were determined by experimental design (maximal models) and model fit diagnostics were assessed using the ‘DHARMa’ package (98). Temperatures include 20°C, 25°C, 30°C and the experimental heatwave (HW, see methods). Model structures are shown using R syntax (additive effect = ‘+’, interaction = ‘*’, zero inflation formula = ‘zi’). Where appropriate the significance of fixed effects was tested using Analysis of Variance (ANOVA type III) using the ‘car’ package (89).

## Data Availability

Raw sequences deposited in the NCBI Sequence Read Archive (SRA) under BioProject: PRJNA1208452, data and code are available at Figshare DOI: 10.25446/oxford.28523561.
