# Supplementary material for: Virulent Parasites Emerge in Hosts With Rising Temperatures
Source: Glob Chang Biol. 2026 Sep 8;32(9):e71082. doi: 10.1111/gcb.71082 (PMC13552160; doi:10.1111/gcb.71082)
Supplement: Supplementary file 1 [file GCB-32-e71082-s001.pdf]

Supporting information

Title: Virulent parasites emerge in hosts with rising temperatures

Authors: Tobias E. Hector[1*], Julia M. Kreiner[2,3], James C. Forward[4], Kim L. Hoang[1,5], Emily J. Stevens[1,6], Serena Johnson[7], Jingdi Li[1,7], Kayla C. King[1,2,4,7*]

Affiliations:

[1.] Department of Biology, University of Oxford; Oxford, UK, OX1 3SZ

[2.] Biodiversity Research Centre, University of British Columbia; Vancouver, BC Canada V6T 1Z4

[3.] Department of Ecology & Evolution, University of Chicago; IL 60637, United States

[4.] Department of Microbiology & Immunology, University of British Columbia; Vancouver, BC Canada

[5.] Emory School of Medicine, Emory University; Atlanta, GA 30322, United States

[6.] School of Life Sciences, Keele University; Staffordshire, UK, ST5 5BG

[7.] Department of Zoology, University of British Columbia; Vancouver, BC Canada

* Corresponding author. Email: kayla.king@ubc.ca; toby.hector.cv@pm.me

Keywords: Climate warming, host-parasite, virulence, population genomics, experimental evolution

Supporting information

Tables

Table S1: Host mortality following exposure to host-evolved populations passaged at constant temperatures relative to the ancestor. Parameter estimates from a linear mixed effect model testing the effects of evolution temperature (20, 25, and 30°C) and assay temperature (20, 25, and 30°C) on mortality for host-evolved parasite populations relative to the average ancestral value. Host-evolved population values were centered on the average ancestral value at each assay temperature and modelled using a zero-intercept linear mixed effect model. The difference from zero of estimated marginal means was assessed using t-tests.

Evolution temperature	Assay temperature	Estimated Marginal Mean	Lower 95% CL	Upper 95% CL	df	t-ratio	p-value
**20**	**20**	-2.366	-5.451	0.720	45	-1.544	0.130
**25**	**20**	-1.379	-4.465	1.706	45	-0.900	0.373
**30**	**20**	1.306	-1.779	4.392	45	0.853	0.399
**20**	**25**	1.352	-1.734	4.437	45	0.882	0.382
**25**	**25**	4.054	0.968	7.140	45	2.646	0.011
**30**	**25**	3.328	0.243	6.414	45	2.173	0.035
**20**	**30**	-6.881	-9.966	-3.795	45	-4.491	<0.001
**25**	**30**	-2.428	-5.513	0.658	45	-1.585	0.120
**30**	**30**	-1.989	-5.075	1.097	45	-1.298	0.201

Table S2: Virulence (host mortality) of parasite populations passaged at constant temperatures during assays across the three constant temperatures (20, 25, and 30°C). Shown are the significance of fixed effect estimated from a model of host mortality, predicted by evolution temperature treatment (levels = "20", "25", and "30°C"), infection treatment (levels = "host-evolved" and "environmental control"), assay temperature (levels = "20", "25", and "30°C"), and all higher order interactions. The data were modelled using a generalized linear mixed effect model (GLMM) with a Beta-binomial (log link) error distribution using the "glmmTMB" package. Fixed effects were estimated with Analysis of variance (ANOVA, type III), using the "car" package.

Term	Chisq	df	p-value
**(Intercept)**	75.761	1	<0.001
**Evo temperature**	35.696	2	<0.001
**Infection treatment**	59.508	1	<0.001
**Assay temperature**	745.626	2	<0.001
**Evo temp:Inf treat**	17.901	2	<0.001
**Evo temp:Assay temp**	0.778	4	0.941
**Inf treat:Assay temp**	2.526	2	0.283
**Evo temp:Inf treat:Assay temp**	1.152	4	0.886

Table S3: Pairwise comparisons between host-evolved and environmental control populations at each level of evolution temperature, averaged across assay temperature due to the lack of higher order interactions. Estimated from the GLMM reported in Table S2. Contrast were calculated on estimates back-transformed to the original response scale and so estimates represent proportional differences. Bonferroni corrected p-values are shown.

Evo temp	Contrast	Estimate	s.e	z-ratio	p-value
**20°C**	Host-evolved vs environmental	0.154	0.028	5.556	<0.001
**25°C**	Host-evolved vs environmental	0.201	0.028	7.108	<0.001
**30°C**	Host-evolved vs environmental	0.040	0.033	1.205	0.685

Table S4: Virulence (host mortality) of parasite populations passaged at 25°C, 30°C, or under the heatwave temperatures during simulated heatwave (25°C - 35°C) assays. Shown are the significance of fixed effect estimated from a model of host mortality, predicted by evolution temperature treatment (levels = "25", "30°C", and Heatwave), infection treatment (levels = host-evolved and environmental control), and their interaction. The data were modelled using a generalized linear mixed effect model (GLMM) with a Beta-binomial (log link) error distribution using the "glmmTMB" package. Fixed effects were estimated with Analysis of variance (ANOVA, type III), using the "car" package.

Term	Chisq	df	p-value
**Evo temp**	0.195	2	<0.001
**Infection treatment**	24.034	1	<0.001
**Evo_temp:inf_treat**	12.856	2	0.002

Table S5: Pairwise comparisons between host-evolved and environmental control populations at each level of evolution temperature during a heatwave assay. Estimated from the GLMM reported in Table S5.

Assay temp	Evo temp	Odds ratio	s.e	z-ratio	p-value
**Heatwave**	25°C	0.428	0.0764	-4.754	<0.0001
	30°C	0.513	0.0943	-3.632	0.0003
	Heatwave	0.998	0.1731	-0.013	0.9892

Table S6: Parameter estimates from a linear mixed effect model testing the effects of evolution temperature (25°C, 30°C, and heatwave) on mortality under heatwave conditions for host-evolved parasite populations relative to the average ancestral value. Host-evolved population values were centered on the average ancestral value and modelled using a zero-intercept linear mixed effect model. The difference of estimated marginal means from zero was assessed using t-tests.

Assay temperature	Evolution temperature	Estimated Marginal Mean	Lower 95% CL	Upper 95% CL	df	t-ratio	p-value
**Heatwave**	25°C	8.34	4.604	12.086	15	4.754	<0.001
	30°C	4.34	0.598	8.080	15	2.472	0.026
	Heatwave	-2.84	-6.577	0.906	15	-1.615	0.1270

Table S7: Significance of fixed effects from a MANOVA testing the effects of evolution temperature (20, 25, 30°C) and assay temperature (20, 25, 30°C) on the relationship between relative host mortality and relative within-host colony forming units (CFU), for host-evolved parasite populations. Relative trait values were produced by subtracting the average environmental control's value from the matching evolution temperature treatment. This relative measure allows the partitioning of changes to phenotypic traits that were specific to within-host processes.

Term	Pillai test statistic	Approx F Value	df	Num d.f.	Den d.f.	p-value
**Evo_temp**	1.256	38.001	2	4	90	<0.001
**Assay_temp**	0.00	0.000	2	4	90	1.000
**Evo_temp:Assay_temp**	0.549	4.256	4	8	90	<0.001

Table S8: Counts of genome-wide *de novo* variants at different levels of minor allele frequency (MAF). Average counts are shown for each experimental evolution treatment, with the standard deviation (SD).

Infection treatment	Evolution temperature	Mean number of SNPS genome wide (SD)	Mean SNPS MAF >0.1 (SD)	Mean SNPS MAF >0.5 (SD)	Mean number of fixed (MAF =1) SNPS (SD)
**Host**	20°C	65.3 (4.97)	51.3 (3.33)	4.5 (0.55)	2.2 (0.75)
	25°C	65.2 (4.26)	51.2 (3.97)	3.8 (0.98)	2.3 (0.52)
	30°C	63.5 (9.77)	50.3 (7.97)	4.2 (1.33)	2.4 (0.55)
	Heatwave	64.83 (8.89)	50.0 (7.72)	4.8 (1.17)	1.4 (0.55)
**Environmental**	20°C	55.33 (9.29)	45.7 (5.78)	4.7 (1.53)	1.3 (0.58)
**control**	25°C	73.0 (7.21)	54.3 (5.03)	8.0 (6.24)	2.7 (0.58
	30°C	100.67 (24.79)	68.7 (15.28)	6.3 (2.89)	2.3 (0.58)
	Heatwave	80.0 (10.0)	63.0 (2.00)	4.3 (1.54)	2.3 (0.58)

Table S9: ANOVA (Type III) of a fully parameterised model showing the significance of fixed effects for the effect of evolution temperature (20, 25, 30°C, and Heatwave), infection treatment (host-evolved and environmental controls), and level of parallel evolution (No. populations) on mean allele frequency of variants within evolution treatments. The number of within-treatment populations each *de novo* variant arose in (No. populations/Scaled reps) was scaled to between zero and one, to account for the differing number of populations across treatments.

Term	Sum sq	df	F-statistic	p.value
**No. populations (scaled)**	0.874	1	29.4589	**<0.001**
**Evo temp**	0.460	3	5.1685	**0.002**
**Host treat**	0.714	1	24.0662	**<0.001**
**Scaled reps:Evo temp**	0.400	3	4.4933	**0.004**
**Scaled reps:Host treat**	0.345	1	11.6257	**<0.001**
**Evo temp:Host treat**	0.300	3	3.3736	**0.018**
**Scaled reps:Evo temp:Host treat**	0.191	3	2.1448	0.093

Infection treatment	Evolution temperature (°C)	Assay temperature (°C)	Fixed effects	Random effects	Model type	Error distribution	R Package	Model notes
Ancestor	NA	20, 25, 30	Assay temp	(1|Assay date)	Generalized linear mixed model	Beta-binomial	glmmTMB (96)	
Ancestor	NA	25, HW	Assay temp * Bacterial species	NA	Linear model	Gaussian	Stats	
Host-evolved	20, 25, 30	20, 25, 30	0 + Evo temp * Assay temp	(1|population)	Linear mixed model	Gaussian	lme4 (95)	Relative to (centered on) ancestral average.
Host-evolved & Environmental controls	20, 25, 30	20, 25, 30	Evo temp * Infection treatment * Assay temp	(1|Assay temp/ population) + (1|Assay date)	Generalized linear mixed model	Beta-binomial	glmmTMB	
Host-evolved & Environmental controls	25, 30, HW	HW	Evo temp * Infection treatment	(1|population)	Generalized linear mixed model	Beta-binomial	glmmTMB	

Table S10: Details of statistical models analysing host mortality or colony forming units (CFU) phenotype data. Each model assessed the effects of experimental "Evolution temperature" treatment, "Infection treatment", "Assay temperature" (temperature at which traits were measured at the end of the experimental evolution), or combinations thereof, as fixed effects. Generalized Linear Mixed Models were fitted using the "glmmTMB" package (96), and Linear Mixed Models using "lme4" package (95). Fixed and random effect structures were determined by experimental design (maximal models) and model fit diagnostics were assessed using the "DHARMa" package (98). Temperatures include 20°C, 25°C, 30°C, and the experimental heatwave (HW, see methods). Model structures are shown using R syntax (additive effect = "+", interaction = "*", nested random effect = "/"). Where appropriate the significance of fixed effects was tested using Analysis of Variance (ANOVA type III) using the "car" package (89).

Infection treatment	Evolution temperature (°C)	Assay temperature (°C)	Fixed effects	Random effects	Model type	Error distribution	R Package	Model notes
Ancestor	NA	20, 25, 30	Assay temp	NA	Kruskal-Wallis rank sum test & Dunn test	Non-parametric	Stats & Rstatix (96)	
Host-evolved & Environmental controls	20, 25, 30	20	Evo temp * Infection treatment	(1|population) + zi(Evo temp * Host treat)	Generalized linear mixed model	Negative Binomial	glmmTMB	
Host-evolved & Environmental controls	20, 25, 30	25	Evo temp * Infection treatment	(1|population)	Generalized linear mixed model	Negative Binomial	glmmTMB	
Host-evolved & Environmental controls	25, 30, HW	30	Evo temp * Infection treatment	(1|population)	Generalized linear mixed model	Poisson	glmmTMB	

Table S11: Details of statistical models analysing colony forming units (CFU) phenotype data. Each model assessed the impact of experimental "Evolution temperature" treatment, "Infection treatment", "Assay temperature" (temperature at which traits were measured at the end of the experimental evolution), or combinations thereof, as fixed effects. Generalized Linear Mixed Models were fitted using the "glmmTMB" package (96), and Linear Mixed Models using "lme4" package (95). Fixed and random effect structures were determined by experimental design (maximal models) and model fit diagnostics were assessed using the "DHARMa" package (98). Temperatures include 20°C, 25°C, 30°C, and the experimental heatwave (HW, see methods). Model structures are shown using R syntax (additive effect = "+", interaction = "*", zero inflation formula = "zi"). Where appropriate the significance of fixed effects was tested using Analysis of Variance (ANOVA type III) using the "car" package (89).

Figures

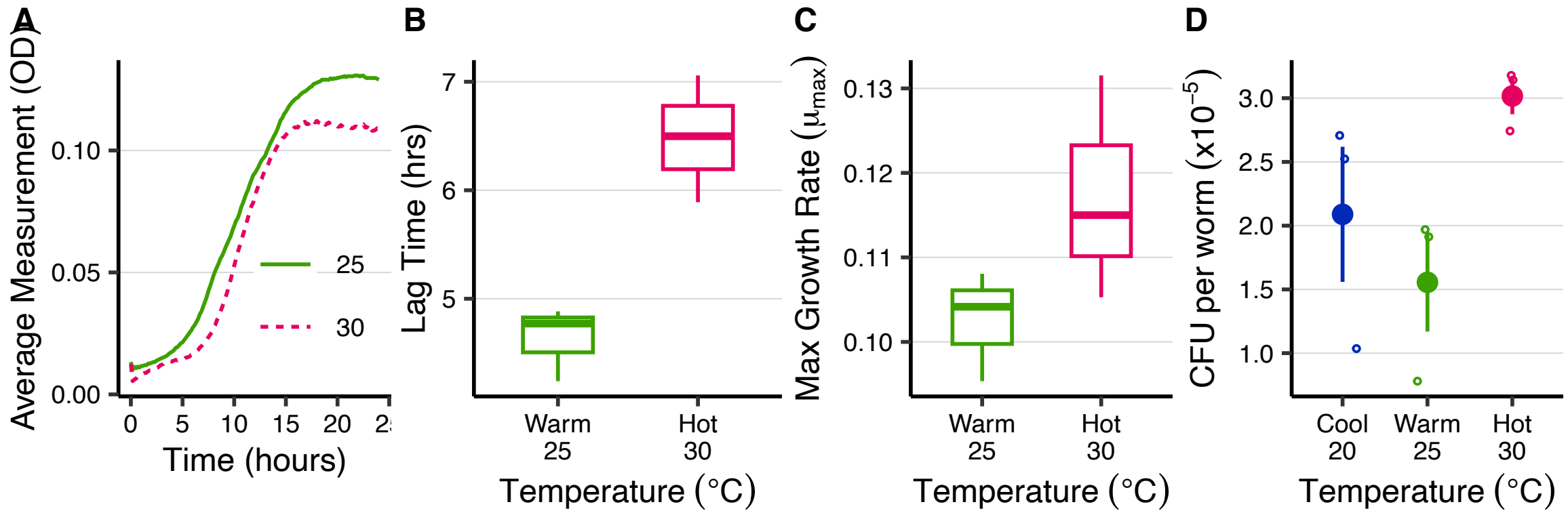

Figure S1: *In vitro* growth metrics and within-host burden for the ancestral parasite clone across temperature. A) Average optical density (OD_{600}) measurements across time for *L. musarum* samples grown at either 25 or 30°C. B) Average lag time at each temperature. C) Average maximum growth rate. Metrics were calculated using the gcplyr R package (79) (see methods, n = 3 per temperature). D) Colony Forming Units (CFU) – a measure of within-host bacterial load – of ancestral parasite isolate across three constant environmental temperatures (± SE). While the effect of temperature here was marginally non-significant (Kruskal-Wallis Test: statistic = 5.956, d.f. = 2, p-value = 0.051), there was a trend towards increased numbers of within-host bacterial cells at 30°C (Dunn Test 25°C-30°C: statistic = 2.385, p-value = 0.017, adj. p-value = 0.0512, n = 3 per temperature).

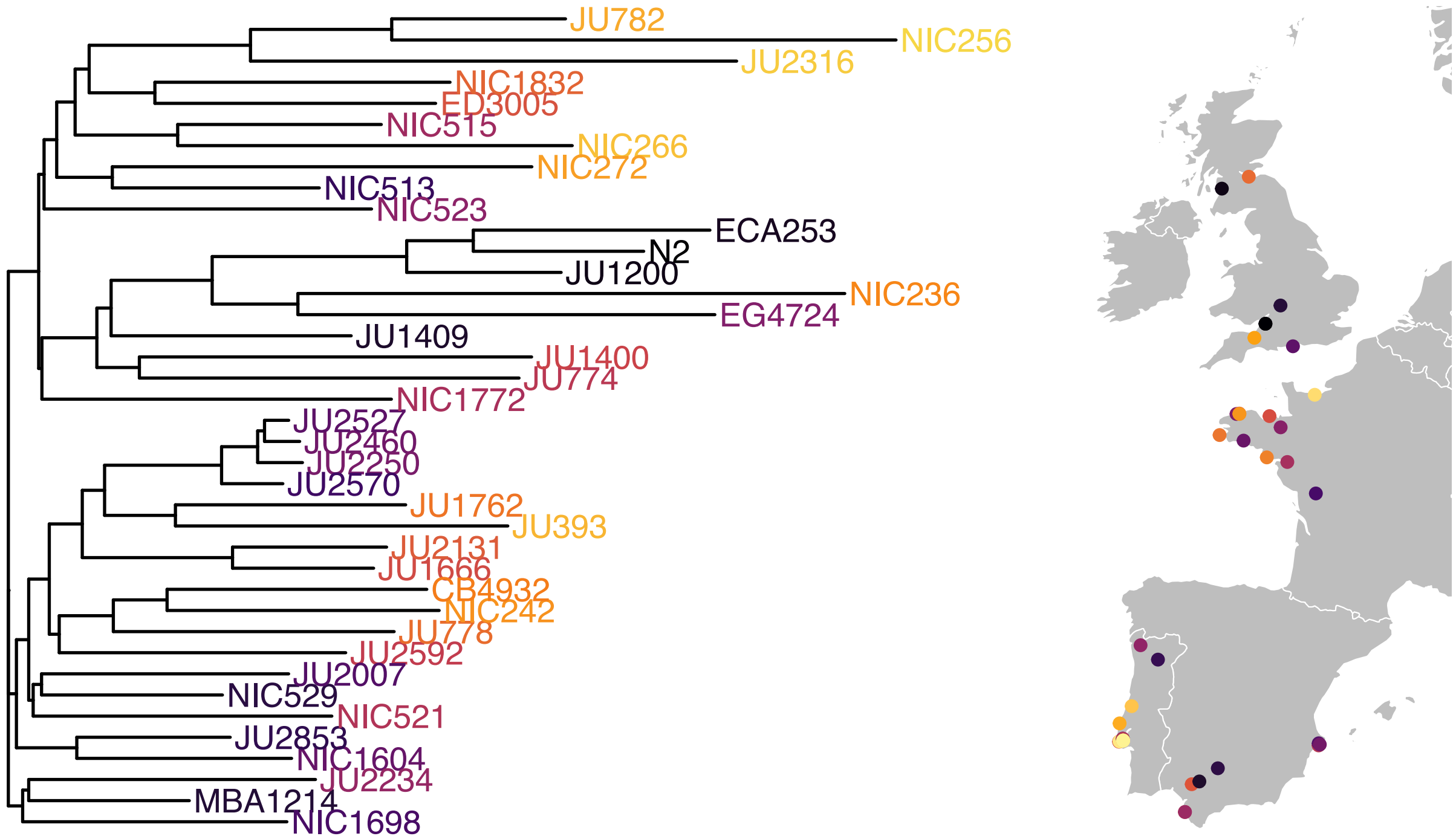

Figure S2: Phylogeny and collection locations for a subset of European *C. elegans* wild-type isolates. Tip label colours and map points represent the genomic distance from the domesticated N2 strain. Data from: Crombie, T. A., McKeown, R., Moya, N. D., Evans, K. S., Widmayer, S. J., LaGrassa, V., Roman, N., Tursunova, O., Zhang, G., Gibson, S. B., Buchanan, C. M., Roberto, N. M., Vieira, R., Tanny, R. E., & Andersen, E. C. (2023). CaeNDR, the Caenorhabditis Natural Diversity Resource. Nucleic Acids Research, gkad887. https://doi.org/10.1093/nar/gkad887

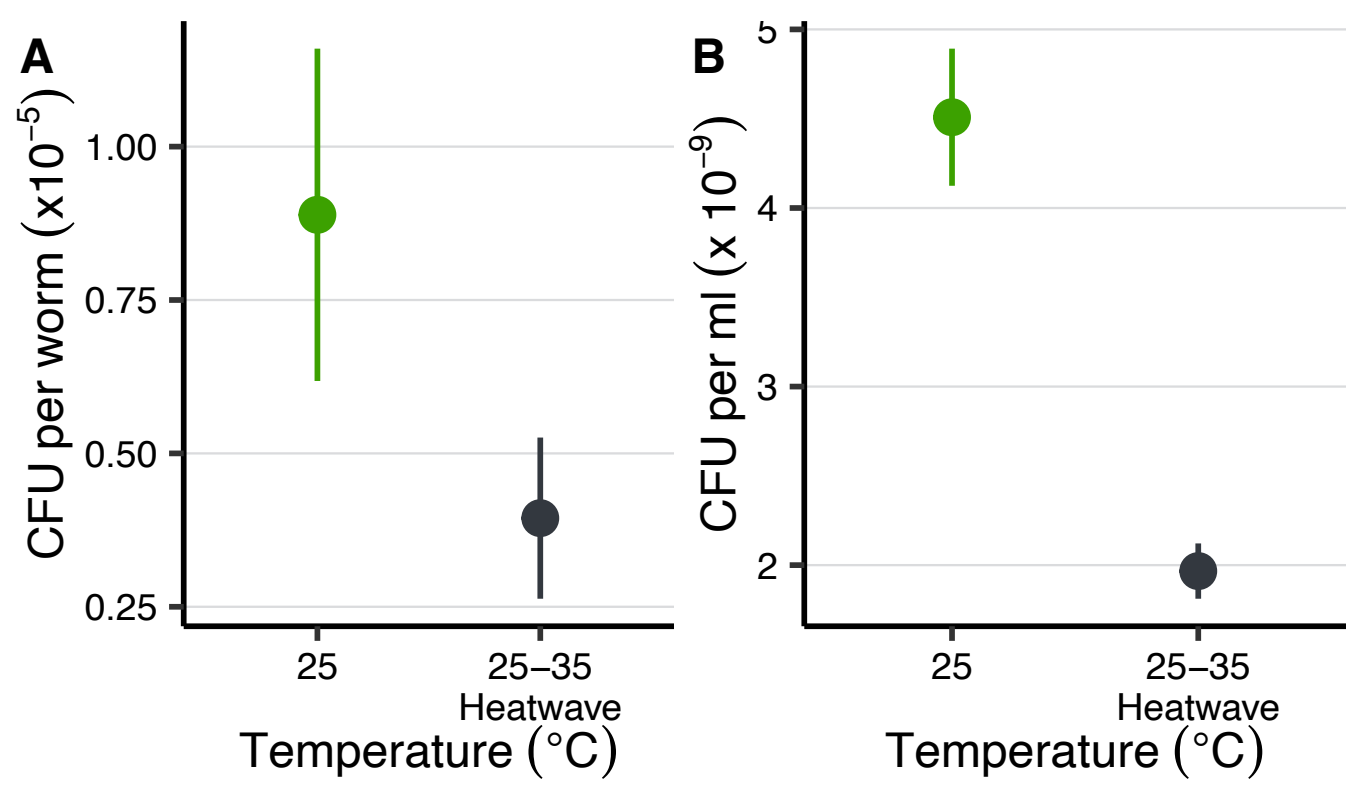

Figure S3: Viability of *L. musarum* following an experimental heatwave. A) *In vivo* cell viability (± SE). Colony forming units within host *C. elegans* after exposures at either 25°C or 35°C heatwave. The trend for reduced CFU following heatwave was not significant (Kruskal-Wallis Test: statistic = 1.191, d.f. = 1, p-value = 0.275, n = 3 per temperature). B) *In vitro* cell viability (± SE). Twelve overnight culture of *L. musarum* grown from individual colonies were distributed evenly across three 96-well plates and maintained at 25°C or exposed to a three-hour experimental heatwave at 35°C. Following the heatwave, all replicates were spotted on LB agar and incubated at 25°C for 48 hours, after which the number of colonies were counted. There were significant fewer viable cells following heatwave (Kruskal-Wallis Test: statistic = 14.552, d.f. = 1, p-value = <0.001, n = 12 per temperature).

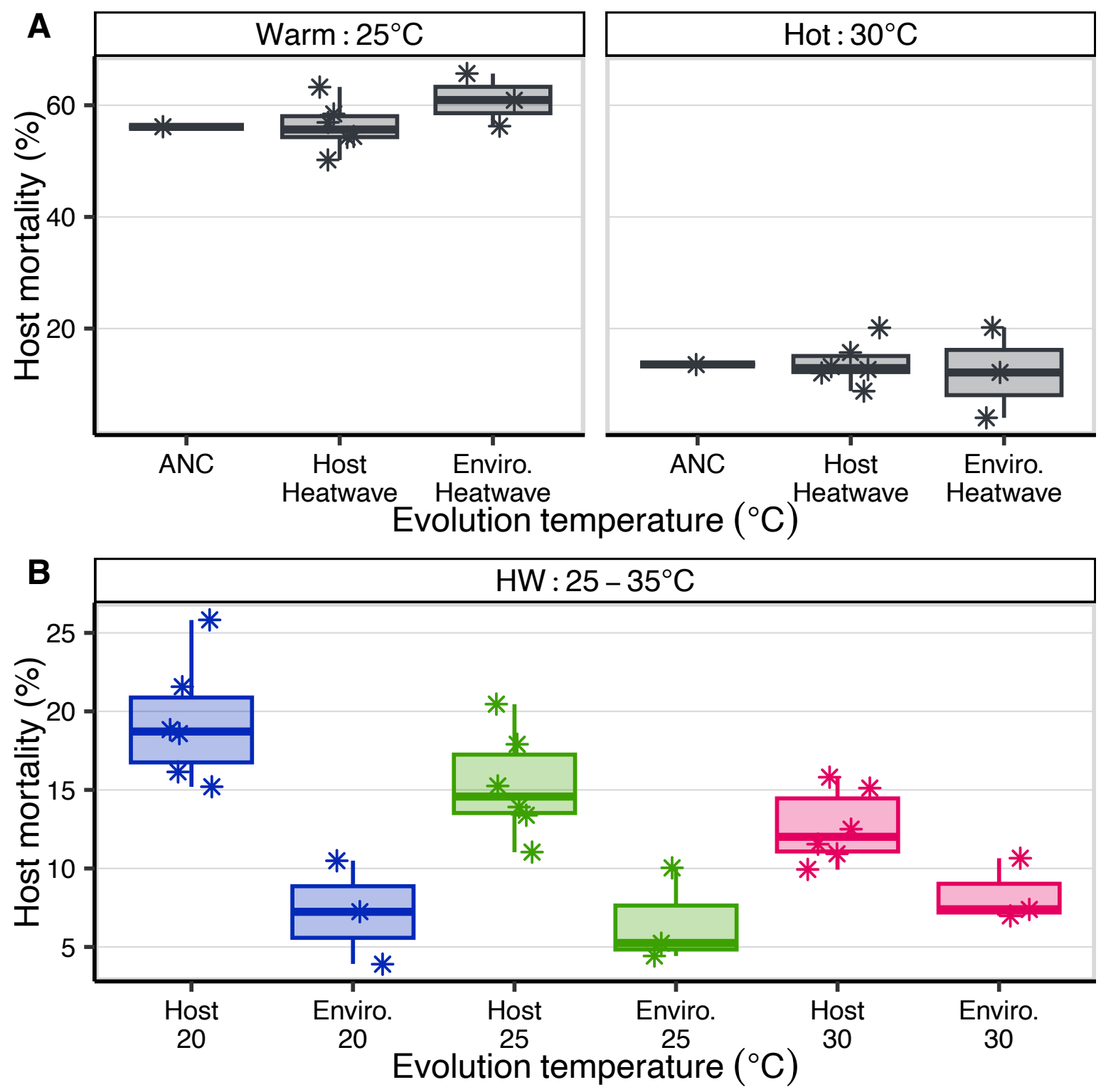

Figure S4: Mortality of *C. elegans* exposed to evolved *L. musarum* populations (or the ancestral isolate). A) Mortality was measured for host-evolved (Host) and environmental control (Enviro.) parasite populations evolved under heatwave (and the ancestral isolate) during assays at constant 25°C or 30°C (facets). B) Mortality of host-evolved and environmental control parasite populations evolved at 20, 25, or 30°C measured during a heatwave assay.

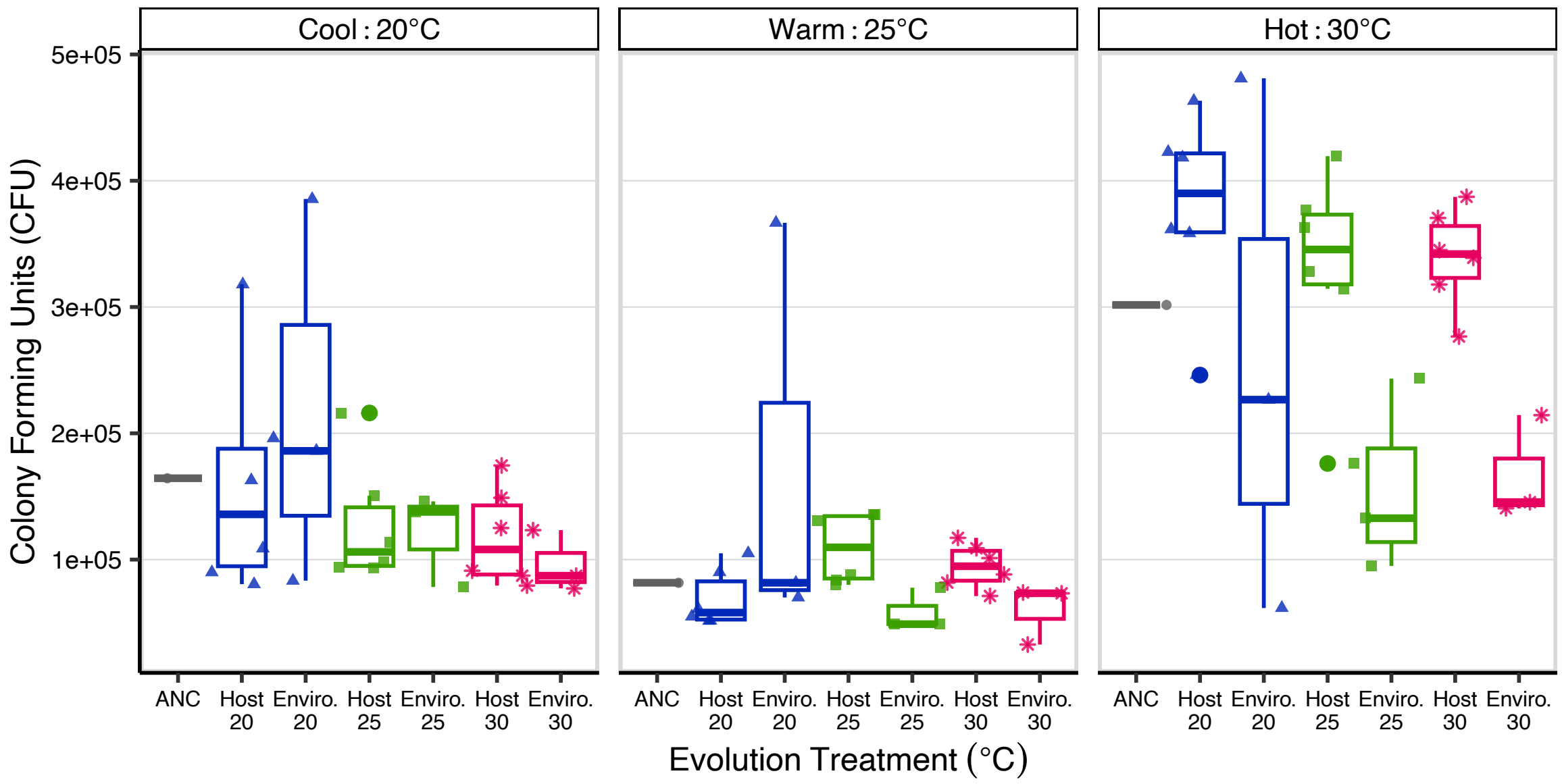

Figure S5: Within-host colony forming units (CFUs) depend on evolution treatment and assay temperature. Within-host CFUs from nematode hosts exposed to the ancestral isolate (ANC), host-evolved, or environmental controls (enviro) populations at either 20, 25, or 30°C. Parasite CFUs of all populations were assayed at each constant temperature (facets). Points are the average of three technical replicates.

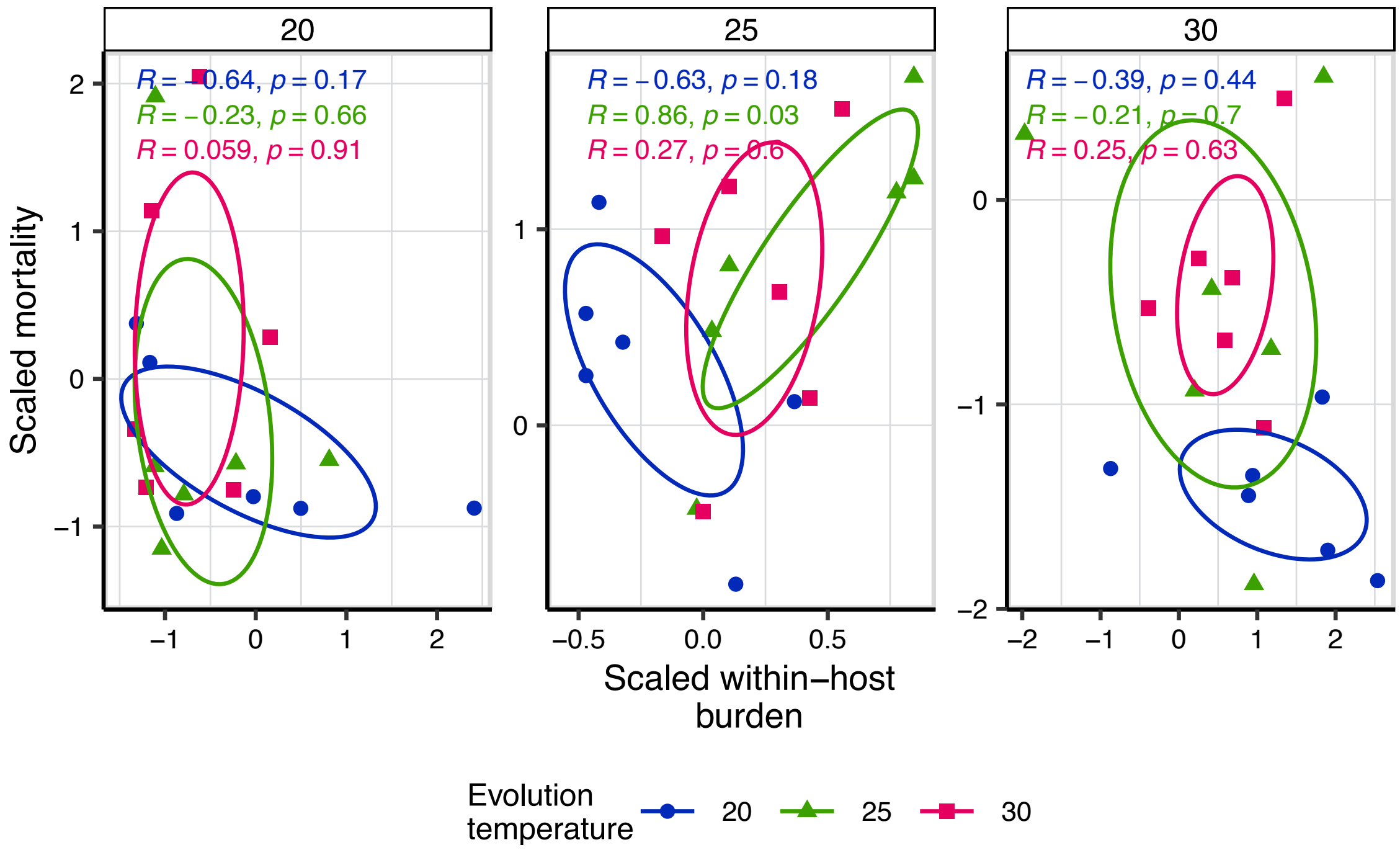

Figure S6: Raw data (scaled, and centered on ancestor) for colony formatting units (within-host burden) and virulence (mortality) of *L. musarum* populations host-evolved at 20, 25, or 30°C. Traits were measured for populations from all treatments at each temperature in subsequent assays (facets). Coloured text shows Pearson's correlation coefficients for each treatment and temperature (20°C = blue, 25°C = green, 30°C = pink). As with the data shown in Main Figure 2C, there is only a positive relationship between within-host burden and host mortality for parasite populations passaged at warm 25°C (n = 6 per treatment combination).

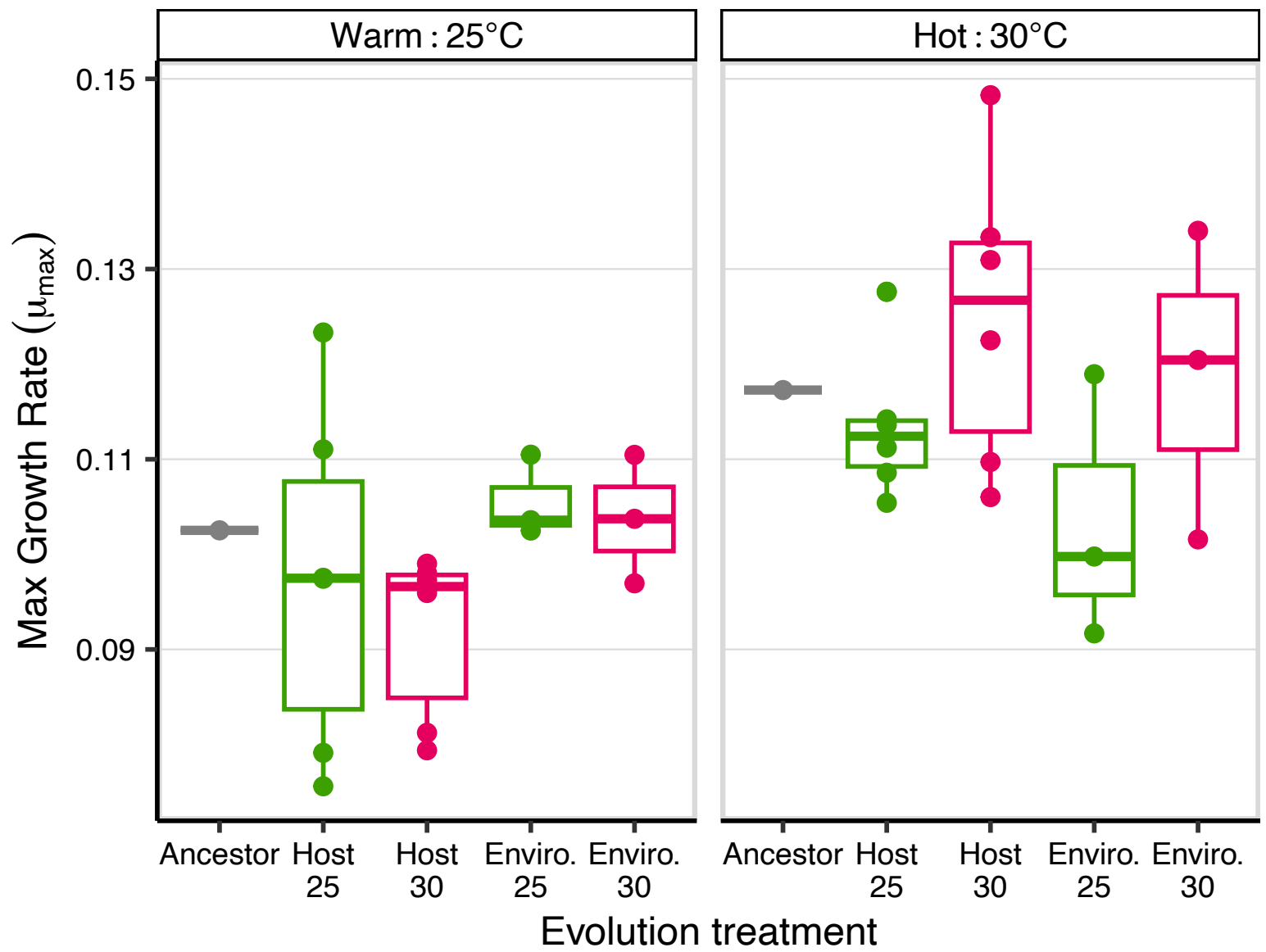

Figure S7: *In vitro* growth rates for parasite populations at two test temperatures (25°C & 30°C). Shown are the calculated maximum growth rates (μ_{max}) for parasite populations or the ancestral isolate during *in vitro* growth in 96-well plates at either 25°C or 30°C (facets). Both host-evolved (Host) and environmental controls (Environ.) are shown.

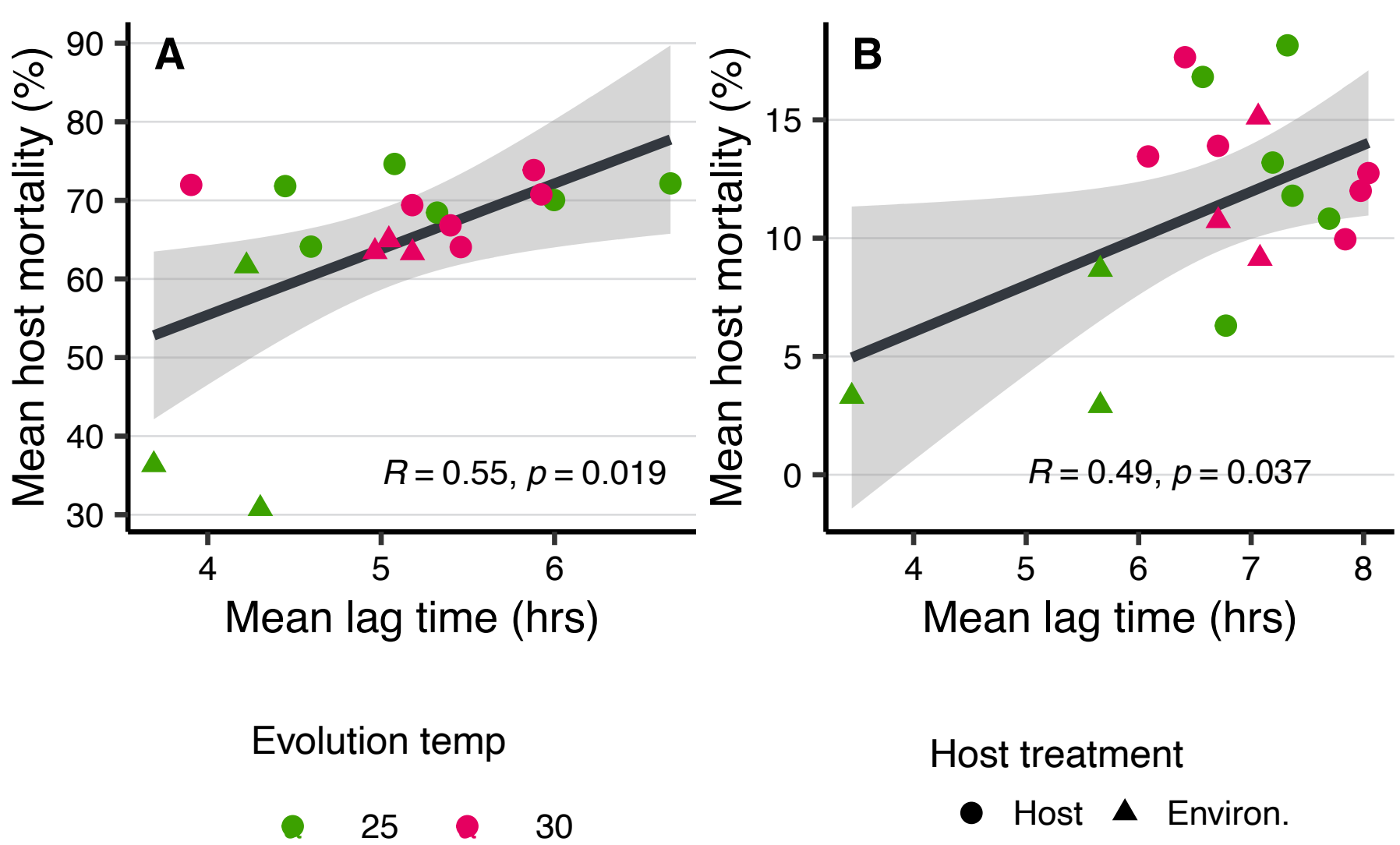

Figure S8: The relationship between mean *in vitro* lag time and mean virulence (host mortality) for each population passaged at 25°C (green) and 30°C (pink) host-evolved (circles) or environmental controls (triangles), at A) 25°C, or B) 30°C assay temperatures. Inset text shows Pearson's correlation coefficients (n = 18).

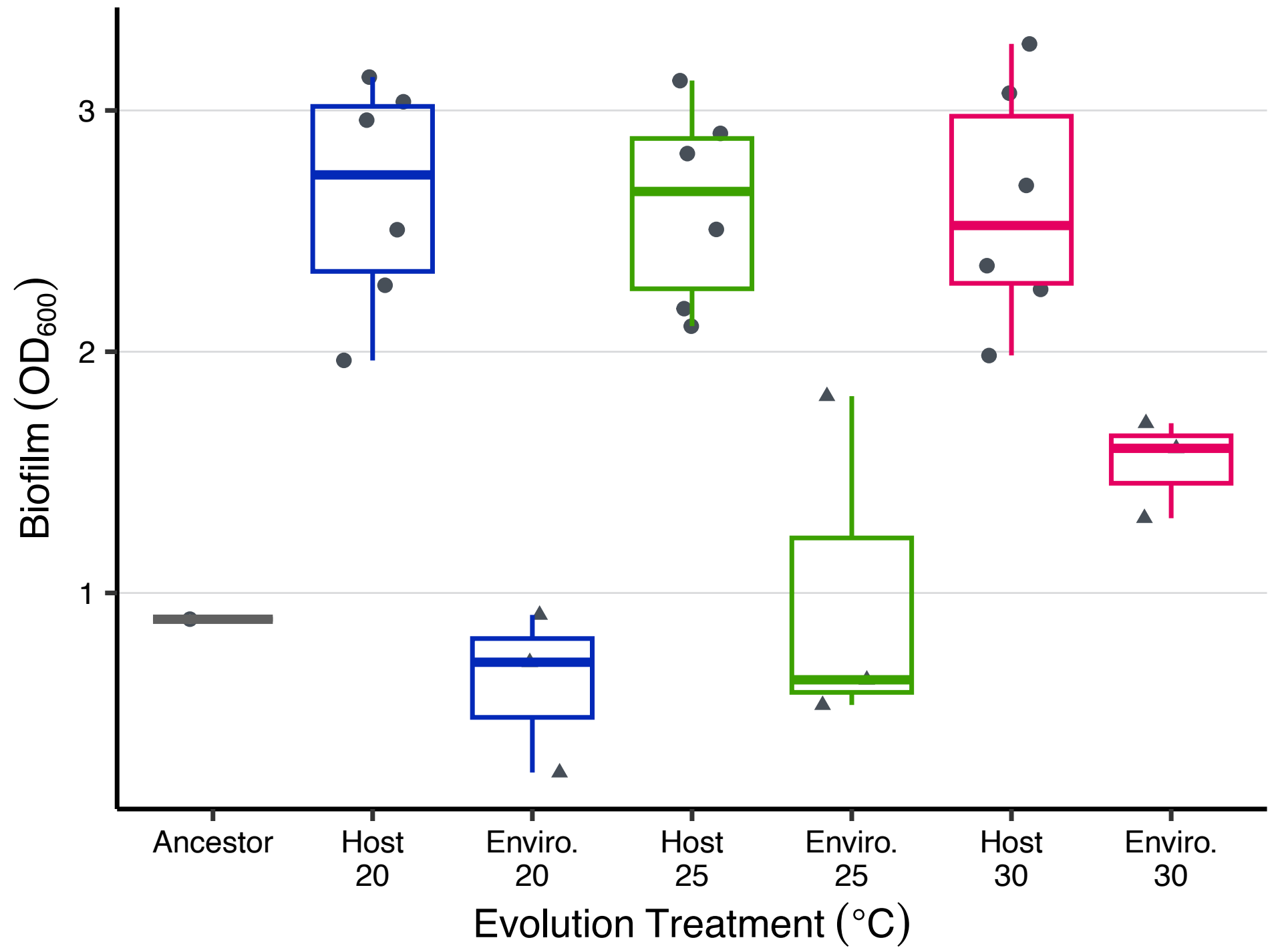

Figure S9: Increased biofilm formation in host-evolved parasite populations. Average biofilm formation (measured as optical density 600nm) for parasite populations and the ancestral isolate. Biofilm formation was measured for populations grown at 25°C. Host = host-evolved treatments, Environ. = environmental controls, ANC = ancestral isolate. Each point is the mean of six technical replicates.

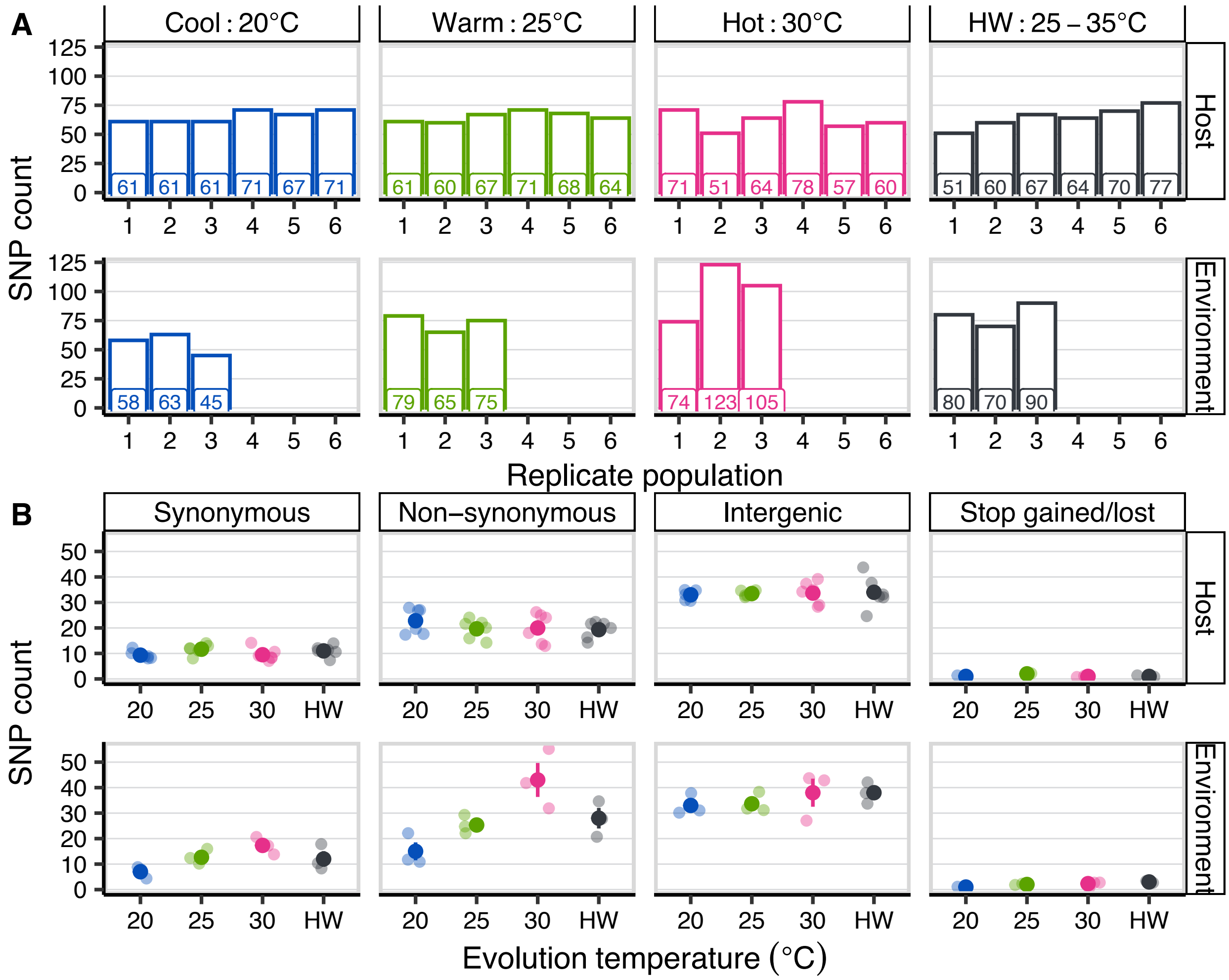

Figure S10: Single nucleotide polymorphism (SNP) counts for parasite populations from each treatment. A) Counts of variants (genome-wide) for each population from pools of 40 sequenced clones. The x-axis shows each independent population. B) Average SNP count (±SE) per treatment for different mutation type. Transparent points show counts for each independent population.

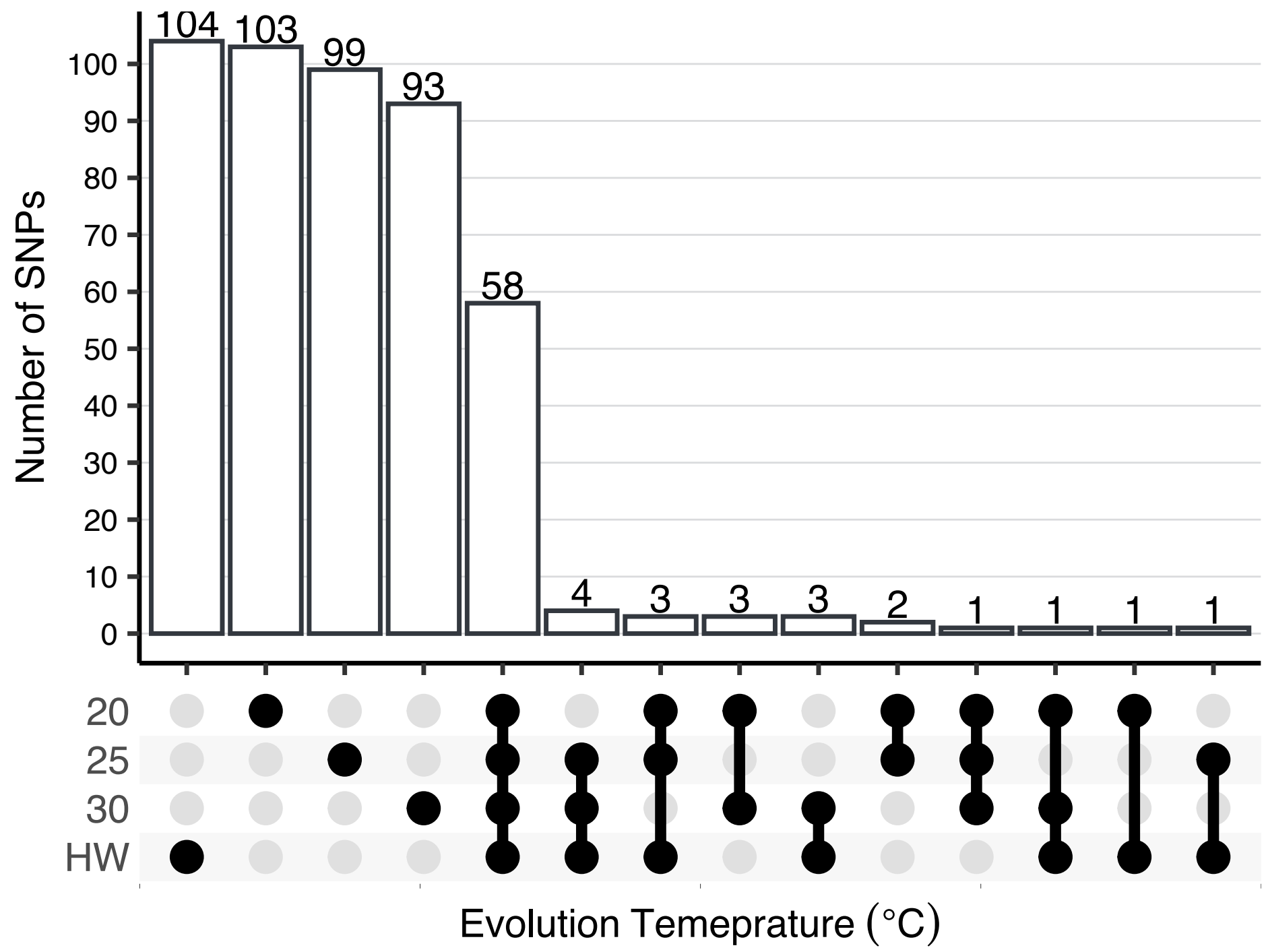

Figure S11: Most single nucleotide polymorphisms (SNPs) are either shared across all treatments or unique to a single treatment. Shown are the number of SNPs (genome-wide) that occurred in within and across different combinations of treatments for host-evolved parasite populations.

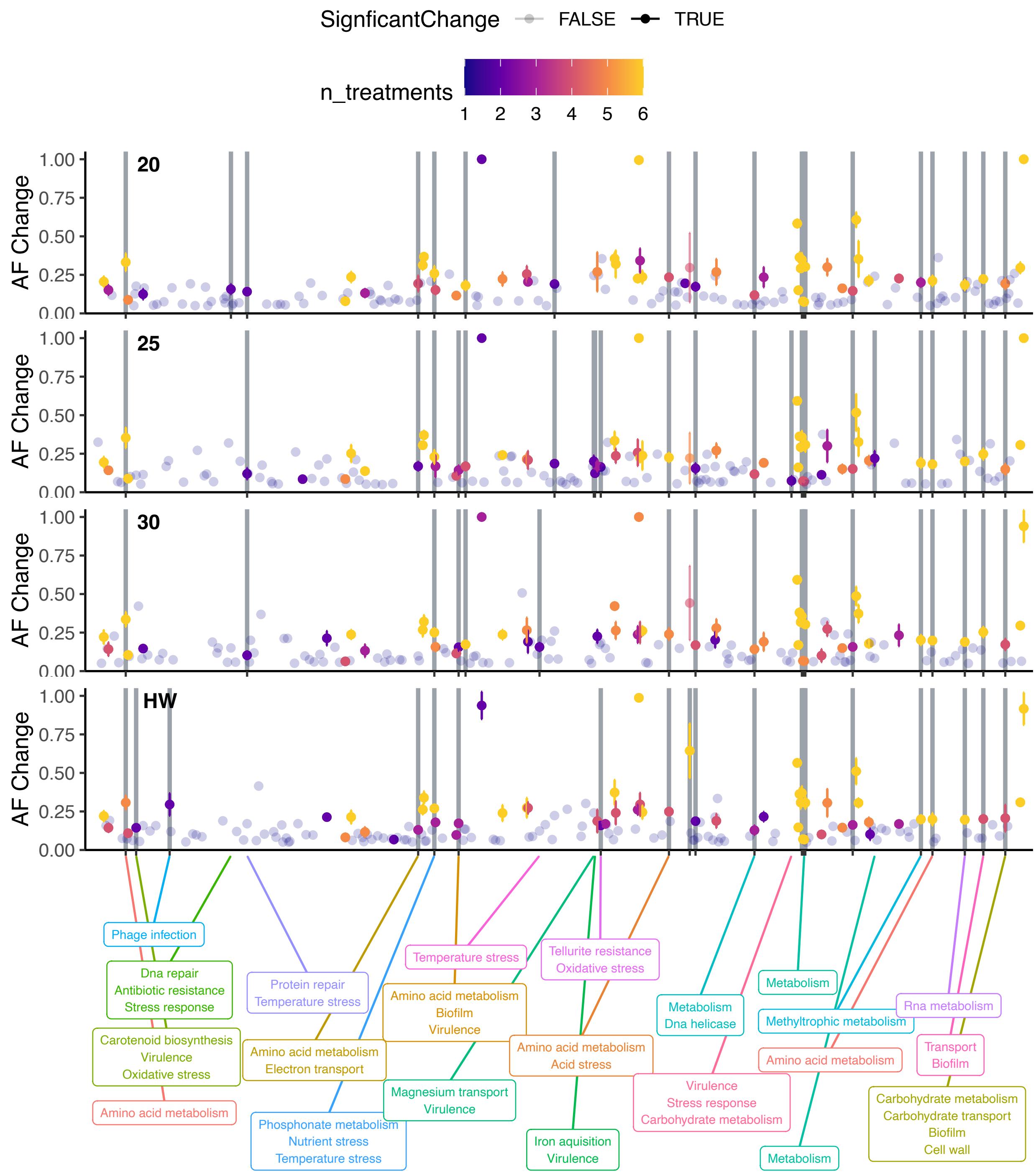

Figure S12: Mean allele frequency change across host-evolved populations from each evolution temperature. Shown are SNPs along the genome. Colour represents the number of populations a SNP arose in parallel, non-translucent points showed significantly higher allele frequencies than would be expected at random in parallel SNPs. Grey bars show the position of non-synonymous genic SNPs which showed significant allele frequency change in parallel across multiple populations (with the corresponding putative functions below).

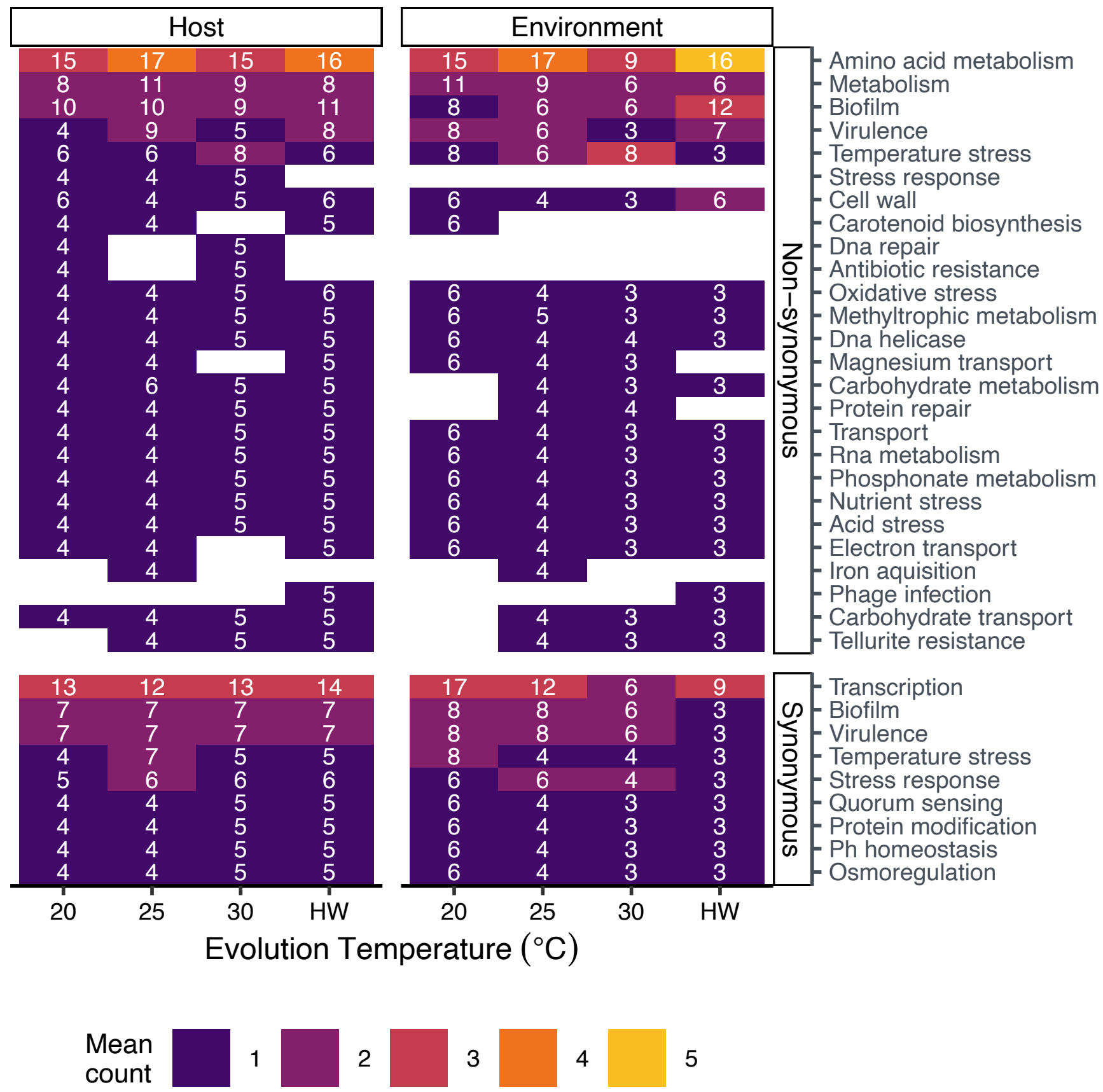

Figure S13: Putative functions and number of genes potentially under selection. For each evolution treatment, shown are the average SNP counts (colours) and average proportion relative to all SNPs in that treatment (numbers) for each putative functional category. Only SNPs within genes are shown. The data was further filtered to only include SNPs with minor allele frequencies >10% or variants which showed significant parallel evolution across populations within treatment.

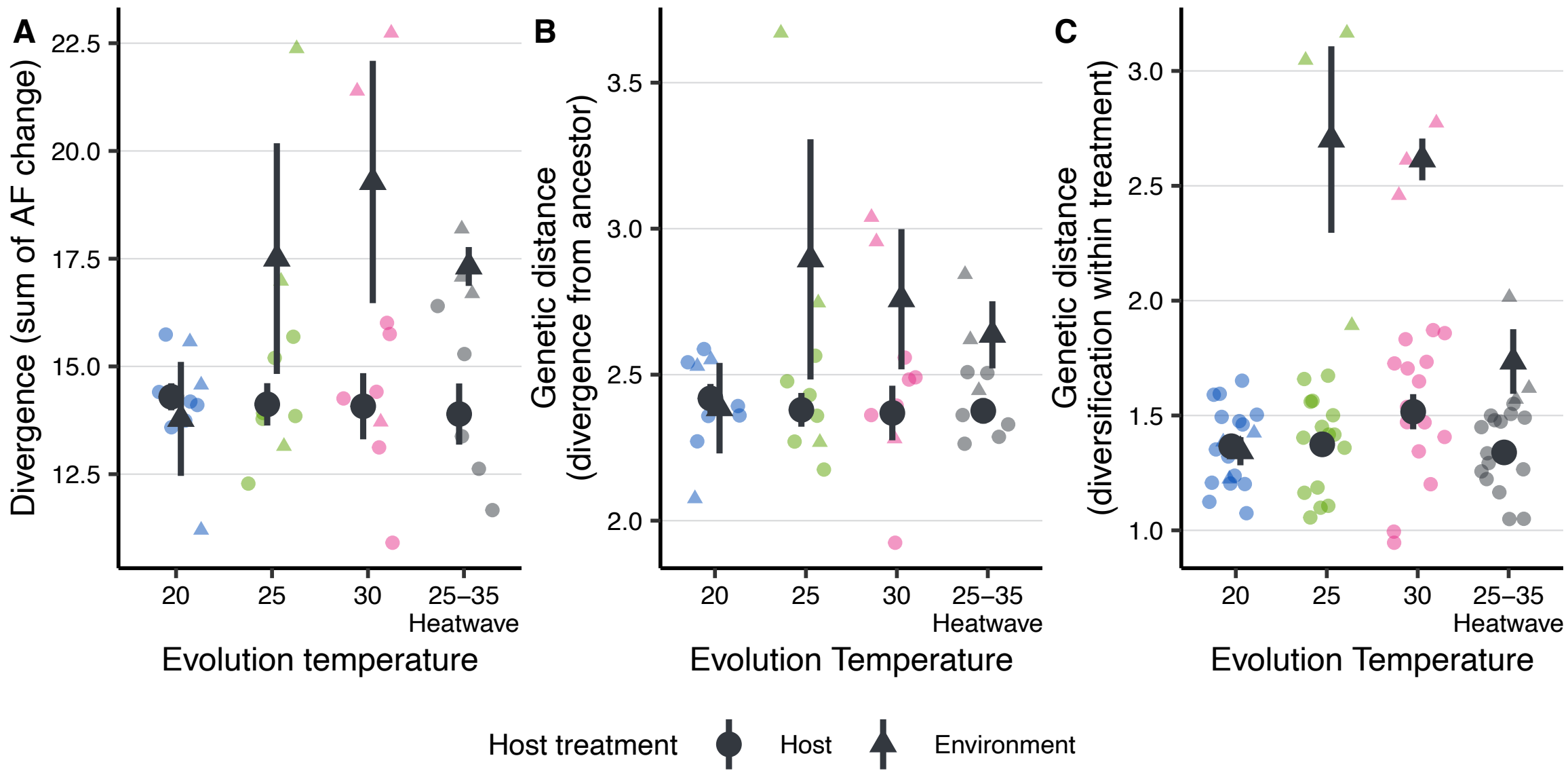

Fig S14: Genetic distance and divergence of host-evolved parasite populations across temperatures. A) Genetic divergence as the sum of allele frequency change for populations in each treatment, B) Euclidean distance of each population to the ancestor from all SNPs (MAF >0.1). C) Euclidean distance between populations within each evolution treatment, showing within treatment diversification. Large black points show the treatment mean (± standard error), coloured points show the population means (A & B) or mean for each specific population-population comparison (C).

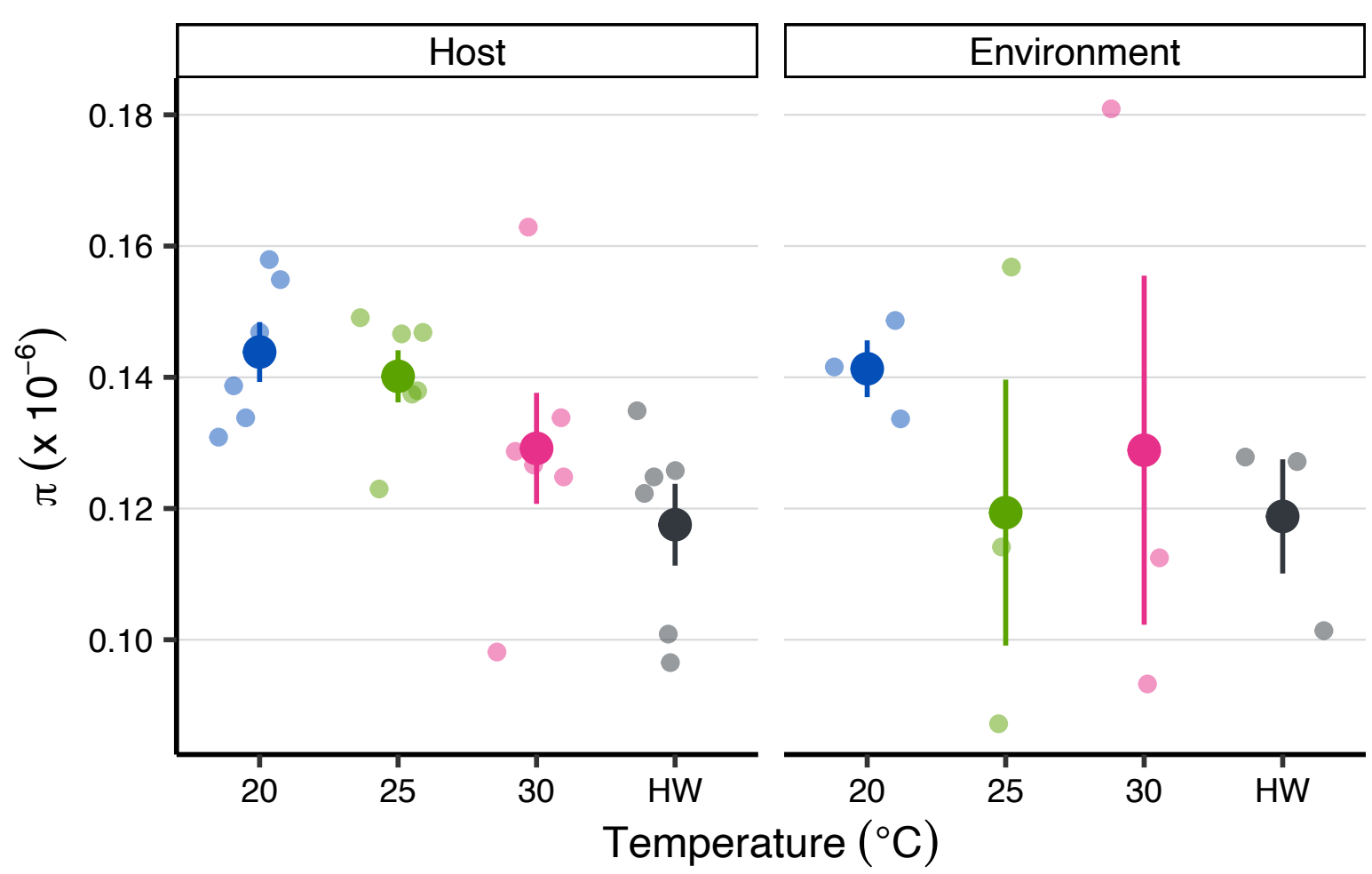

Figure S15: Nucleotide diversity for host-evolved parasite populations declines at the hottest temperature treatments. Per gene nucleotide diversity, predicted using the PoPoolation2 software (87) following the standard workflow (https://sourceforge.net/p/popoolation2/wiki/Main/). Data shows the mean (± SE) of the average genome-wide nucleotide diversity of populations within each treatment.

Appendix: Detailed experimental protocols

Leucobacter musarum infection assay schedule

Day 1:
- Chunk nematodes to 9cm OP50 NGM plates

Day 4:
- Bleach nematodes
- Seed 9cm NGM plates with OP50 (enough for ~2000 worms per plate)

Day 5:
- Count L1's, and plate on OP50 NGM
- Brew OP50 and parasite from frozen stock

Day 6:
- Prepare experimental exposure plates

Day 7:
- Expose adult worms on exposure plates

Day 8:
- Mortality counts or CFU's

Day 10:
- For CFU assay count colonies

Media and chemicals

Nematode Growth Medium - NGM (1L)

3g NaCLl
17g agar
2.5g peptone
975ml dH_20
1ml 5mg/ml cholesterol
(dissolved in ethanol)
autoclave and allow to cool (to about 50-60° in water bath or hand holding temp.)
1ml 1M $CaCl_2$
1ml 1M $MgSO_4$
25ml 1M Potassium Phosphate
(1L potassium phosphate, pH 6.0 = 868ml 1M KH_2PO_4 + 132ml 1M K_2HPO_4)
pour plates using sterile technique.

LB agar media

Follow instructions on bottle
16g LB powder
14g Agar powder
800ml dH_20
autoclave and allow to cool (to about 50-60° in water bath or hand holding temp).
pour plates using sterile technique.

LB liquid media

20g LB powder
1L dH_20
autoclave and allow to cool

1M $CaCl_2$

14.7g $CaCl_2.2H_20$
100ml dH_20
Autoclave

5mg/ml Cholesterol

0.25g cholesterol
50ml ethanol

1M $MgSO_4$

24.65g $MgSO_4$
100ml dH_20
autoclave

1M KH_2PO_4

136.09g KH_2PO_4
1L dH_20
autoclave

1M K_2HPO_4

87.1g K_2HPO_4
500ml dH_20
autoclave

M9 (1L)

3g KH_2PO_4
6g Na_2HPO_4
5g NaCl
1L dH_20
autoclave and allow to cool (to less than 60° in water bath or hand holding temp.)
1ml $MgSO_4$
If you want to add Triton-x (to make M9-Tx) for worm work add:
1ml 10% Triton-x (in dH_20)

5M NaOH (100ml)

20g NaOH
80ml dH_20
20ml Mix (swill) until cool (it is an exothermic reaction). Then add more dH_20 up to 100ml

Protocols

All protocols assume sterile technique and should be done in either the biological safety cabinet or under the flame unless otherwise stated.

Freezing worms

1. Chunk worms 3-4+ days before freezing
2. Wash starving worms off plates (dauer L2 worms freeze best) using M9 with/without Triton-x into Falcon tubes (see "washing worms off plates")
3. Bleach worms and allow L1s to hatch overnight (see bleaching below)

4. Add 750ul of Worm Freezing Solution to labelled cryovials.
5. Add 500ul of L1s then invert tubes to mix.
6. Put tubes into a polystyrene box and put into the -80°C freezer (so that they freeze slowly).

Thawing frozen worms

1. Take a cryotube from the -80°C freezer. Let it warm to room temperature gradually.
2. Shake tube and Pour/pipette the contents onto OP50 seeded NGM (Some protocols require worms to be put onto unseeded OP50).
3. Let the liquid dry in the biological safety cabinet.
4. Parafilm the plates to ensure they don't get contaminated and pace them in the 20°C incubator.
5. In about a week you should see plenty of worms and can chunk.

Selecting bacterial clones

1. Streak bacteria onto agar plate (LB if OP50 or Leucobacter) using inoculation loop.
2. Streak as the image below shows: change inoculation loop between streak 1 and 2.
3. Place this plate at 30°C overnight (OP50) or 25°C for 48 hours (*L. musarum*).
4. Pick one colony with an inoculation loop.
5. Swill the picked colony with the inoculation loop into liquid LB media (see "Growing bacterial cultures")

Growing bacterial cultures

1. Use inoculation loops to swab frozen samples from cryotubes or a colony from a LB plate (see "selecting bacterial clones") into LB broth. Use the biosafety hood and sterile technique!
2. Grow OP50 *E. coli* overnight shaking at 30°C (150-200 rpm). ~30ml in 50ml falcon tube.
3. Grow *L. musarum* overnight shaking at 25°C (50-150rpm). 5-6ml LB in a 15ml falcon tube, arrange the tubes in the shaking incubator on their sides, instead of upright, to increase aeration.
4. To check for contamination in OP50, streak overnight cultures on XLD plates and grow overnight at 30°C. To check for contamination in *L. musarum* cultures, streak overnight cultures on LB plates and grow for 48 hours at 25°C. *L. musarum* colonies are well defined, small, yellowish, and 'sticky'.

Freezing bacteria

1. 500µl of 50% glycerol into each cryotube
2. Add 500µl of overnight bacterial culture into each cryotube then invert tubes to mix
3. Store samples in -80°C freezer.

To 'seed' NGM with E. coli OP50

1. Pipette 100-200µl of OP50 culture onto 9cm NGM plate.
2. Use a L-shaped plate spreader to spread the bacteria.
3. Grow lawns at 30°C overnight.

Making experimental parasite 'exposure' plates

For experimental evolution we used 90mm plates, and for assays we used 60mm and 35mm plates.

1. Grow OP50 and *L. musarum* cultures in liquid culture overnight as described above. Worms don't eat *L. musarum*, so we create a mix *L. musarum* and OP50 when exposing worms.
2. Measure the OD_{630} of all OP50 and *L. musarum* cultures, and dilute:
    a. *L. musarum* = OD_{630} 0.2
    b. OP50 = OD_{630} 0.4

To calculate volumes needed to dilute use the formula C1V1 = C2V2. For 6cm exposure plates, you will want about 100µl of bacterial culture per plate, so work out what final volume of bacteria you will need to seed all exposure plates.

3. Create a *L. musarum*:OP50 mixture with your diluted cultures, with 20% *L. musarum* and 80% OP50
4. Vortex *L. musarum*:OP50 cocktail to mix (it is ok to vortex *L. musarum* once it is very dilute)
5. Pipette 200µl (90mm plates), 100µl (60mm plates), or 50µl (35 plates) of the *L. musarum*:OP50 mixture onto each NGM plate and spread with a spreader covering most of the plate
6. Incubate overnight at 25°C

Washing worms off plates

1. Add 5ml M9-Tx to a worm plate.
2. Swill the liquid around and gently use L-shaped spreader to dislodge the worms.
3. If you are not worried about disturbing the bacterial lawn or if you are trying to also get eggs use the pipette to "pressure wash" the worms off (they can stick to the plate).
4. Tilt the plate to allow worms to sink to one edge, then pipette wormy M9 into a labelled falcon tube (careful not to suck up the agar)

Bleaching worms

Only bleach plates where you can see eggs and gravid hermaphrodites (ones with eggs in them) to ensure success!

Wash worms off your plates using M9 (see "washing worms off plates"). Use a sterile L-shaped spreader to scrape the surface of plates to dislodge eggs.

Pipette wormy M9 into 14ml falcon tubes so that there is 4ml in each tube.

I sometimes combine worms from multiple plates into single falcons (3 plates max).

If you have washed multiple plates into each falcon tube, centrifuge the tubes at 3500 for 2 minutes, and pipette off supernatant, leaving 4ml in each tube.

In a separate falcon tube, mix 50:50 ratio of bleach (10-12% sodium hypochlorite) and 5M NaOH.

You want enough 50:50 bleach NaOH mixture to be able to add at least 1ml to each of your worm falcon tubes.

Make sure the sodium hypochlorite is fresh (it degrades in light over weeks) but also not too strong. Dilute bleach 50:50 with water or M9 if it is too strong.

Add 1ml of the bleach NaOH solution to each 4ml worm sample.

Shake/vortex regularly for 7-8 minutes.

At first, all the worms go really straight, like sticks. Then they usually tend to bend in the middle (in hermaphrodites, this is where the vulva is and so is quite easily penetrated by the solution). The liquid in the tube should also become less cloudy. If it does not the bleach may be too weak.

After 7-8 minutes vortex the tubes for up to 1 minute.

Place the falcon tubes in the centrifuge and spin for 2 minutes at 3500rpm.

Remove the supernatant (leaving the pellet). Pipette in 5ml M9. Centrifuge again

Remove the supernatant (leaving the pellet). Pipette in 5ml M9. Centrifuge again

After this second wash, remove the supernatant (leaving the pellet). Pipette 3ml M9 into each tube and vortex to re-suspend the pellet.

To achieve L1 arrest shake put the tubes overnight in the 20°C.

Some research suggests long periods in M9 is not great for worms, however.

Plating L1's on OP50

Take tubes from 20°C.

If your wormy M9 has lots of dead animals (after bleaching):

allow the sample to stand in a falcon rack for a minute or so to allow most of the large dead worms to settled to the bottom. Don't wait too long or too many L1's will settle as well.

After a minute or so, pipette most of the supernatant into a new falcon, leaving the majority of the dead worms in the old falcon.

Count the number of worms per μl of your sample:

Briefly vortex the sample so that all the worms are homogenous through the sample.

Pipette six 2μl droplets of the sample onto an empty petri dish. Count the number of worms present under the microscope.

Calculate the average of the number of worms per μl.

Calculate the number of μls you need to put onto each plate (this depends on the amount of worms you want on each plate. 1000-2000 L1's per 9cm plate).

E.g. I want to plate 1000 L1 worms and I counted 10 worms per μl: 1000/10 = 100μl per plate.

It is good to avoid plating high volumes of liquid. If there are very few L1's per μl, they can be concentrated by centrifuging at 1600rpm for 1 minute. Remove some supernatant, vortex and then re-count.

At this point you can work out whether you have enough worms for your experiment by looking at the total volume of wormy M9 you have.

Pipette calculated volume onto NGM plates seeded with OP50 (see "To seed NGM"). Between plates regularly vortex/shake sample to keep the worms distributed evenly in the sample.

Allow the plates to dry in the biological safety cabinet and put them in the incubator of choice (usually 20°C if growing worms for an experiment).

Exposing worms to parasite

Wash worms off your OP50 plates using M9 (see "washing worms off plates").

If you expect that there will be a low number of worms per μl, concentrate sample by centrifuging at 1600rpm for 1 minute, and remove a some of the supernatant.

Count the number of worms per μl of your sample:

Briefly vortex the sample so that all the worms are homogenous through the sample.

Pipette six 2μl droplets of the sample onto an empty petri dish. Count the number of worms present under the microscope.

Calculate the average of the number of worms per μl.

Calculate the number of µls you need to put onto each plate (this depends on the amount of worms you want on each plate). Re-concentrate worms if needed to plate the smallest volume that is reasonable.

E.g. I want to expose 100 worms per plate and I counted four worms per µl: 100/4 = 25µl per plate.

At this point you can work out whether you have enough worms for your experiment by looking at the total volume of wormy M9 you have.

Pipette this amount of the sample onto each plate that you have previously prepared (see “making exposure plates”). Do this whilst continually shaking the sample to keep the worms distributed evenly in the sample.

Allow the plates to dry in the biological safety cabinet and put them in the incubator of choice (this depends on your experiment).

If there are still dead worms in your sample from the bleaching process use a marker pen to circle around the area where you spotted the worms. When you come to count mortality you can avoid counting any worms within this area to avoid counting worms that died prior to the assay. If the volume plated is as small as possible, the area that needs to be avoided should also be small.

Counting mortality

It’s best to count mortality without knowing the treatments you are counting- code the plates so you don’t unknowingly bias your data.

Draw lines on the bottom of the plate or the lid of a spare plate, splitting the plate into sections of 4/5.

Go across the plate and count the total number of worms visible (dead and alive) using a hand clicker. Note this down. Use the segments on the plate as guides- count one section before moving on to the next). Doing it at reasonable speed is best as worms move!

Go back through each section and now count the number of dead worms. Poke them with a platinum wire to see if they move if you are unsure if they are alive or not.

Divide the number of dead by the total number of worms.

Washing worms

Use a platinum wire to pick up the worms from exposure plates and place them into a 1.5ml Eppendorf tube filled with 1200µl M9 with Triton-x. Check the pick carefully to make sure all worms have gone into the tube.

Centrifuge tube(s) at 2000rpm for 1 minute.

Remove 1000µl of the supernatant, being careful not to suck up any worms.

Pipette 1000µl M9-X into tubes.

Centrifuge at 2000rpm for 1 minute

Remove 1000µl of the supernatant, being careful not to suck up any worms.

Pipette 1000µl M9-X into tubes.

Centrifuge at 2000rpm for 1 minute

Remove 1000µl of the supernatant, being careful not to suck up any worms.

Pipette 1000µl M9 (without Triton-x) into tubes.

Centrifuge at 2000rpm for 1 minute

Remove 1000µl of the supernatant, being careful not to suck up any worms.

The worms will now be reasonably clean

Colony forming units (CFU) of bacteria in worms

Prepare one Eppendorf tube (2ml if using Qiagen Tissue Lyser) per replicate with exactly 1200µl M9-x and pour in around 10 sterile beads. Be as sterile as possible.

Under the microscope pick 10 worms per replicate (i.e. plate) into the Eppis.

Do this with a flame on to keep the Eppis sterile while they are open and to flame the picker. Check the pick under the microscope to make sure all the worms have been transferred to the M9. Flame the picker between each replicate to sterilise.

Once all replicates are picked, wash them according to "Eppendorf" method (see above).

After the final wash step you want exactly 200µl of M9 along with your worms left in the tubes.

Crush the worms with a tissue lyser.

Bead beater settings: Speed depends on which one you use, but do max speed for 3-8 minutes. After crushing the worms, hold the tubes up to a light and gently flick the tubes – you will be able to see worms floating about if the crush hasn't worked.

Create a dilution series of your crushed sample in a 96-well plate using M9 that does NOT contain triton-x.

Use a multichannel pipette to put 180µl of M9 into each well.

In the first row of wells, pipette 20µl of each neat sample (this first well will be a 10-1 dilution).

Use the multichannel pipette to then make a serial dilution down to 10-4 by pipetting 20µl of each dilution into the subsequent one. Mix each dilution by aspirating the pipette up and down 3 times as you dispense the sample.

Change tips between each dilution.

Plate between three and six 10-20µl spots of multiple dilutions (for *L. musarum* 10-3 & 10-4 are often best) on 90mm LB plates. Make sure plates are labelled well so you know which the sample ID and dilution of each set of spots. You should be able to fit between two and four samples/dilutioons per 90mm LB plate.

Allow to dry in the sterile cabinet, then put plates at 25°C for 48 hours or until you can see distinct colonies.

Once colonies are visible, they can be counted straight away, or plates can be put at 4°C and counted later.

Use a clicker or colony counter to count the number of colonies. Using a marker pen you can mark each colony as you count to keep track. Don't forget to calculate CFU per worm from your colony counts!

Equation:

CFU per worm=((colony count*dilution factor)*((initial sample volume)/(plating volume)))/(worm number)

E.g. CFU per worm=((colony count*10^(-4))*(200ul/(20ul*6 spots)))/(10 worms)
